# Pd^II^_2_L_4_-type coordination cages up to three nanometers in size†
†Electronic supplementary information (ESI) available: Synthetic procedures and experimental details. CCDC 1511090–1511096. For ESI and crystallographic data in CIF or other electronic format see DOI: 10.1039/c6sc04732g



**DOI:** 10.1039/c6sc04732g

**Published:** 2016-11-18

**Authors:** Suzanne M. Jansze, Matthew D. Wise, Anna V. Vologzhanina, Rosario Scopelliti, Kay Severin

**Affiliations:** a Institut des Sciences et Ingénierie Chimiques , Ecole Polytechnique Fédérale de Lausanne (EPFL) , 1015 Lausanne , Switzerland . Email: kay.severin@epfl.ch; b Nesmeyanov Institute of Organoelement Compounds of the Russian Academy of Sciences , 119991 Moscow , Russia

## Abstract

The utilization of large ligands in coordination-based self-assembly represents an attractive strategy for the construction of supramolecular assemblies more than two nanometers in size. However, the implementation of this strategy is hampered by the fact that the preparation of such ligands often requires substantial synthetic effort. Herein, we describe a simple one-step protocol, which allows large bipyridyl ligands with a bent shape to be synthesized from easily accessible and/or commercially available starting materials. The ligands were used to construct Pd^II^_2_L_4_-type coordination cages of unprecedented size. Furthermore, we provide evidence that these cages may be stabilized by close intramolecular packing of lipophilic ligand side chains. Packing effects of this kind are frequently encountered in protein assemblies, but they are seldom used as a design element in metallasupramolecular chemistry.

## Abstract 2

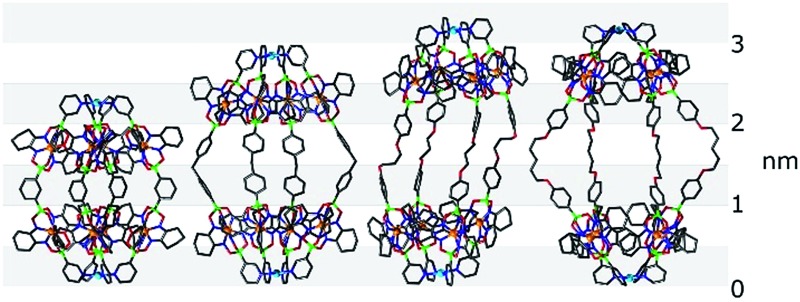
The utilization of easily accessible metalloligands allows the construction of Pd^II^_2_L_4_-type coordination cages of unprecedented size.

## Introduction

Coordination-driven self-assembly provides a formidable means to construct molecularly defined nanostructures through a bottom-up approach.^1^ Ever larger and more complex metallasupramolecular structures have been reported in recent years, including topologically interesting architectures such as rotaxanes, catenanes, knots and links.^2^ Current efforts in this area are increasingly focused on creating metal-based nanostructures with novel functions – such as coordination cages that act as selective catalysts for organic reactions.^3^ However, addressing the ongoing structural challenges presented by coordination-driven self-assembly is similarly important, given the fundamental influence that the structure of a metallasupramolecular architecture exerts over its function. One such challenge is the creation of structurally defined assemblies of low symmetry starting from multiple chemically distinct building blocks (*e.g.* three different metal ions and three different ligands). Various strategies, such as orthogonal self-assembly,^4^ have been employed to tackle this problem, but there remains scope for further development. Another, deceptively simple, challenge is size. In order to create large (>2 nm), molecularly defined nanostructures by self-assembly, one has two options: (a) the assembly of a large number of small building blocks; or (b) the assembly of a small number of large building blocks.^5^

Option (a) is hampered by the fact that multicomponent metallasupramolecular assemblies are typically disfavored from an entropic point of view. Furthermore, intra- and intermolecular interactions must be precisely controlled in order to prepare an assembly of defined stoichiometry and geometry. The latter point is illustrated in recent work by the group of Fujita. They were able to synthesize a M_30_L_60_ coordination cage with a remarkable diameter of more than 8 nm.^6^ In order to avoid alternative structures and insoluble precipitates they had to fine-tune the geometry, solubility and flexibility of the ligand. The work of Fujita also highlights other difficulties in preparing large assemblies by coordination-driven self-assembly: the formation of kinetically trapped intermediates and the presence of large, solvent-filled voids.^6^ These voids are not only problematic for structural analysis by X-ray crystallography, but they can also affect the structural integrity of the assembly upon solvent removal during isolation.^7,8^

Option (b), the utilization of large building blocks, presents further challenges. First and foremost, the synthesis of such molecules can be highly demanding.^9^ We have begun addressing this issue by developing facile synthetic routes for long (up to 5 nm), rigid rod-type metalloligands.^10,11^ Continuing these efforts, we have now prepared a new set of bent metalloligands with terminal 3-pyridyl groups. These ligands were used to form Pd^II^_2_L_4_ complexes, which are significantly larger than the Pd^II^_2_L_4_ cages described to date. Structural and spectroscopic data suggest that some of the assemblies are stabilized by close intramolecular packing of lipophilic ligand side chains. Such packing effects may offer a general means for circumventing the ‘void problem’ outlined above, and to stabilize metallasupramolecular assemblies in highly polar solvents.

## Results and discussion

**Scheme 1:**
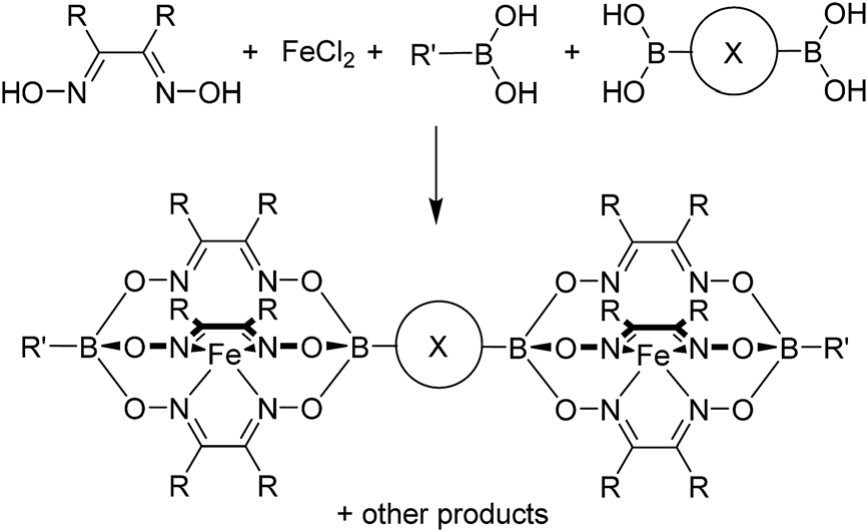
Synthesis of double clathrochelate complexes by combination of a monoboronic acid and a diboronic acid.

Clathrochelate complexes with arylboronate ester caps have been used as metalloligands for the formation of supramolecular assemblies.^12,13^ The synthesis of these ligands can be achieved by a metal-templated condensation reaction of a dioxime and an arylboronic acid,^12,14^ where different functional groups can be introduced by using an appropriate boronic acid or by performing post-synthetic cross-coupling reactions.^15^ Iron(ii) is particularly suited as metal template because the resulting complexes are robust and diamagnetic. An interesting aspect of this chemistry is the fact that it is synthetically straightforward to prepare double clathrochelate complexes by using a mixture of a (functionalized) monoboronic acid and a diboronic acid (Scheme 1). Since the two boronic acids are incorporated in a statistical manner the double clathrochelate complex is formed along with side products (*e.g.* the single clathrochelate complex with two capping groups B–R′). However, purification can be accomplished by chromatography or crystallization. We have previously used this synthetic approach to make linear metalloligands with terminal 4-pyridyl groups.^10^ For the present work, we have focused on bent ligands.

Bent, ‘banana-shaped’ ligands with terminal pyridyl groups are well suited for the construction of Pd^II^_2_L_4_ coordination cages.^1e,f^ Cages of this type have been investigated extensively in recent years. They display interesting host–guest chemistry^1e,f,16^ and undergo self-catenation reactions to yield complex interlocked structures.^17,18^ In addition, they have been studied in the context of medicinal inorganic chemistry.^19^ In order to prepare cages with large cavities, relatively long and rigid ligands are required. Unsurprisingly, the synthesis of such ligands often requires substantial effort.^1e,f^ We hypothesized that the utilization of double clathrochelate complexes as ligands would allow Pd^II^_2_L_4_ cages, with unprecedented size, to be accessed in a straightforward manner.

**Fig. 1:**
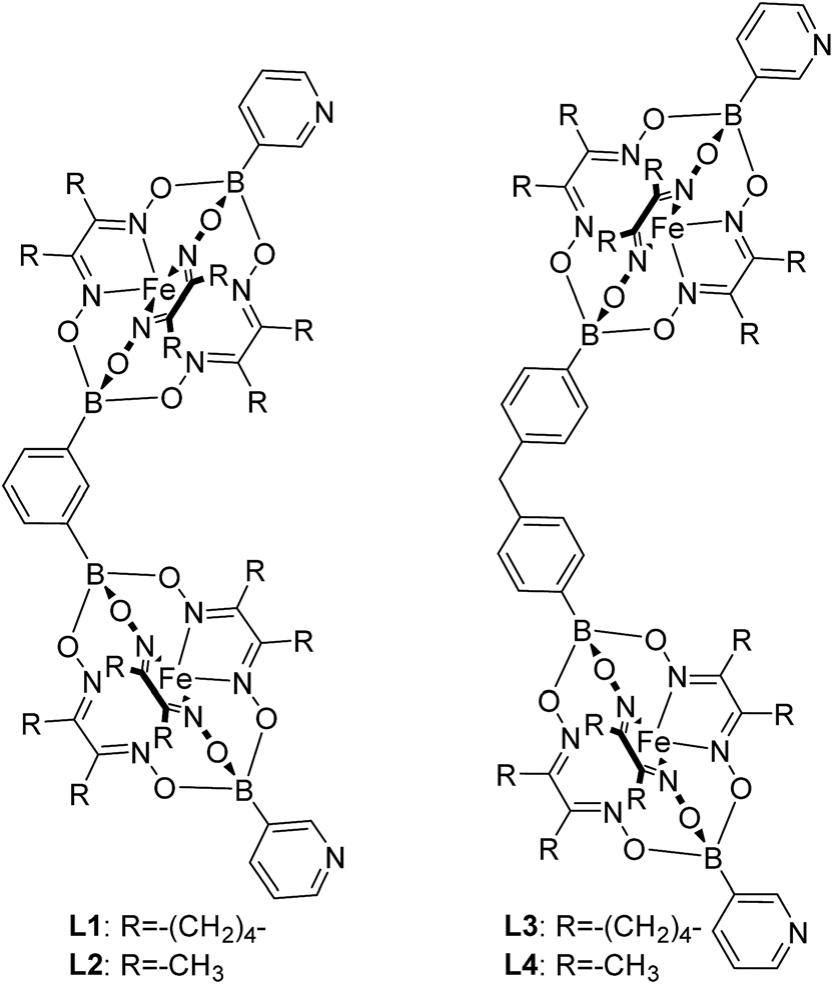
The metalloligands **L1–L4**.

Using the approach outlined in Scheme 1, we have prepared the double clathrochelate complexes **L1–L4** (Fig. 1) by reaction of FeCl_2_ with dimethylglyoxime or nioxime in the presence of 3-pyridylboronic acid and either 1,3-benzenediboronic acid or (methylenebis(1,4-phenylene))diboronic acid. In order to suppress the formation of longer oligomers, we have used substoichiometric amounts of the diboronic acid. The small amount of oligomeric material that is still formed was removed by purification over a short silica plug.

**Fig. 2:**
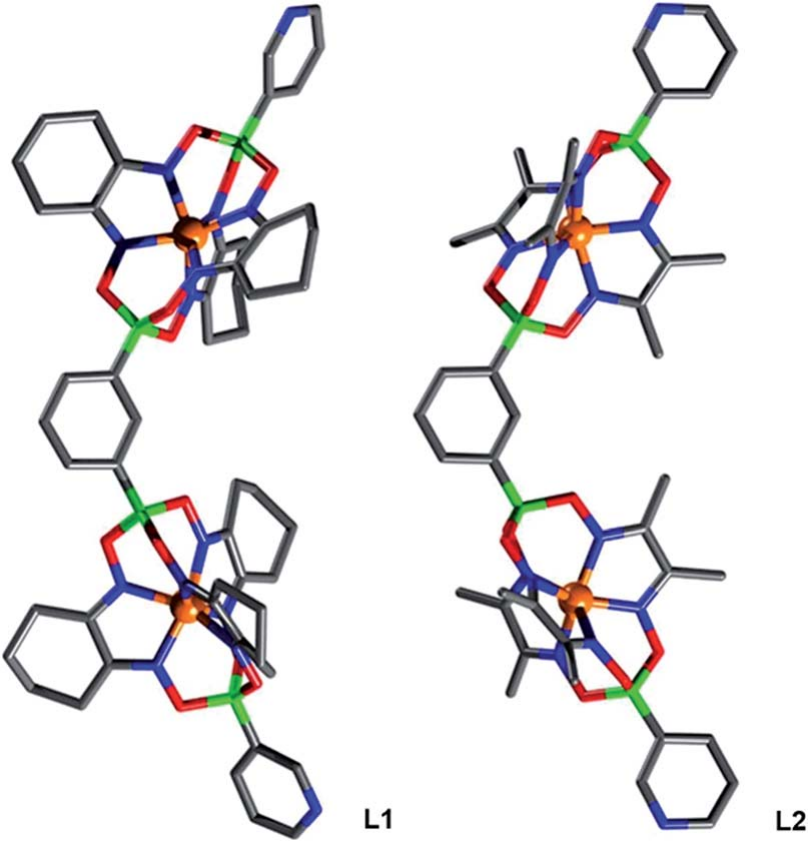
Molecular structures of the double clathrochelates **L1** (left) and **L2** (right) as determined by single crystal X-ray diffraction. Hydrogen atoms and solvent molecules are omitted for clarity. Grey: C; blue: N; green: B; red: O; and orange: Fe.

The main side product—the single clathrochelate complex with terminal 3-pyridyl groups—was separated out by size exclusion chromatography. This one-pot reaction protocol provides rapid access to the double clathrochelate metalloligands **L1–L4** in yields between 39% and 59% (calculated based on the diboronic acid starting material). The new metalloligands were characterized by NMR spectroscopy and high-resolution mass spectrometry (ESI, Fig. S11–S18†). In addition, we have analyzed the solid state structures of **L1** and **L2** by single crystal X-ray crystallography (Fig. 2). The ligands show the expected bent structure and the Fe complexes display the usual distorted trigonal prismatic coordination geometry.

**Scheme 2:**
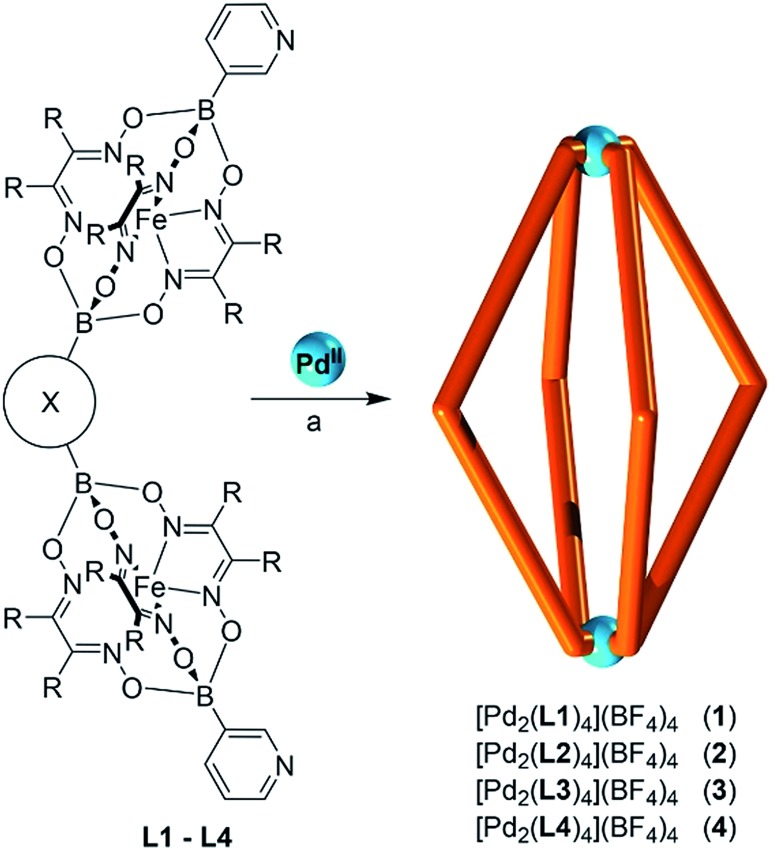
Synthesis of the coordination cages **1–4**. Conditions: (a) [Pd(CH_3_CN)_4_](BF_4_)_2_ (0.5 eq.), CH_3_CN or DMSO, 70 °C, 17 h.

The Pd^II^_2_L_4_ cages **1–4** were prepared by addition of [Pd(CH_3_CN)_4_](BF_4_)_2_ to a suspension of the respective metalloligand in either CD_3_CN or DMSO-*d*_6_ (Scheme 2). The mixtures were then heated at 70 °C for 17 h, resulting in the formation of clear, deep orange solutions. The formation of Pd^II^_2_L_4_ cages was evidenced by NMR spectroscopy and high-resolution mass spectrometry (ESI, Fig. S23–S39†). The ^1^H NMR spectra of the solutions (ESI, Fig. S23–S35†) showed a single set of signals for the aromatic protons of the terminal 3-pyridyl groups. The proton signals belonging to the dioximato ligands gave rise to more complex signal patterns, indicating restricted rotational freedom of the clathrochelate cores (for the free ligands, rotation around the B···Fe···B axis is fast on the NMR time scale). For solutions of complex **1** in CD_3_CN (but not in DMSO-*d*_6_), we also observed a reduced symmetry for the four central phenylene spacers (ESI, Fig S25†). Broadening of the ^1^H NMR signals of the C_6_H_4_ group was observed at 328 K, indicating coalescence (ESI, Fig. S27†). These data provided initial evidence that the cages adopt a compact structure with close intraligand interactions.

DOSY NMR spectroscopy confirmed the formation of aggregates with a defined diffusion coefficient for all four reactions (ESI, Fig. S24, S26, S31, S33, and S35†). The high resolution ESI mass spectra of solutions of **2–4** showed dominant peaks for [Pd_2_L_4_]^4+^ complexes (ESI, Fig. S37–S39†). For cage **1**, on the other hand, a strong peak corresponding to [Pd_2_(**L1**)_4_(BF_4_)]^3+^ was observed (ESI, Fig. S36†), indicating that one of the four BF_4_^–^ anions is more tightly bound. The strong binding of one BF_4_^–^ was corroborated by ^19^F NMR spectroscopy (ESI, Fig. S29†). Two signals were observed, suggesting that the exchange of bound and unbound BF_4_^–^ anions is slow on the NMR time scale.

**Fig. 3:**
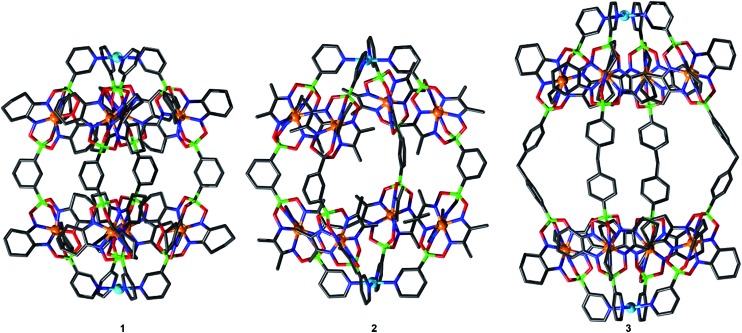
Molecular structures of cages **1** (left), **2** (middle), and **3** (right), as determined by single crystal X-ray diffraction. Hydrogen atoms, solvent molecules and anions are omitted for clarity. Grey: C; dark blue: N; green: B; red: O; light blue: Pd and orange: Fe.

Single crystal X-ray analyses were performed for cages **1**, **2** and **3** (Fig. 3), revealing the expected Pd^II^_2_L_4_ composition. These assemblies adopt a rugby ball-like, prolate-spheroid shape, with Pd(pyridyl)_4_ complexes at the antipodes of the principal axis. With Pd···Pd distances of 2.1 nm (**1**), 2.0 nm (**2**), and 2.7 nm (**3**), the cages are very large compared to the Pd^II^_2_L_4_ cages described so far (to the best of our knowledge, the largest crystallographically characterized Pd^II^_2_L_4_ cage has a Pd···Pd distance of 1.7 nm).^7*c*^ Most of the anions and the co-crystallized solvent molecules in the structures of **1**, **2** and **3** are highly disordered, and the SQUEEZE algorithm was applied to solvent molecules during refinement. For complex **1**, however, we were able to locate one BF_4_^–^ anion inside the cage (ESI, Fig. S46†). The anion is tightly encapsulated within **1**, which is in line with the slow exchange kinetics observed by NMR spectroscopy (ESI, Fig. S29†) and the MS data (ESI, Fig. S36†) showing that one counter anion is retained.

**Fig. 4:**
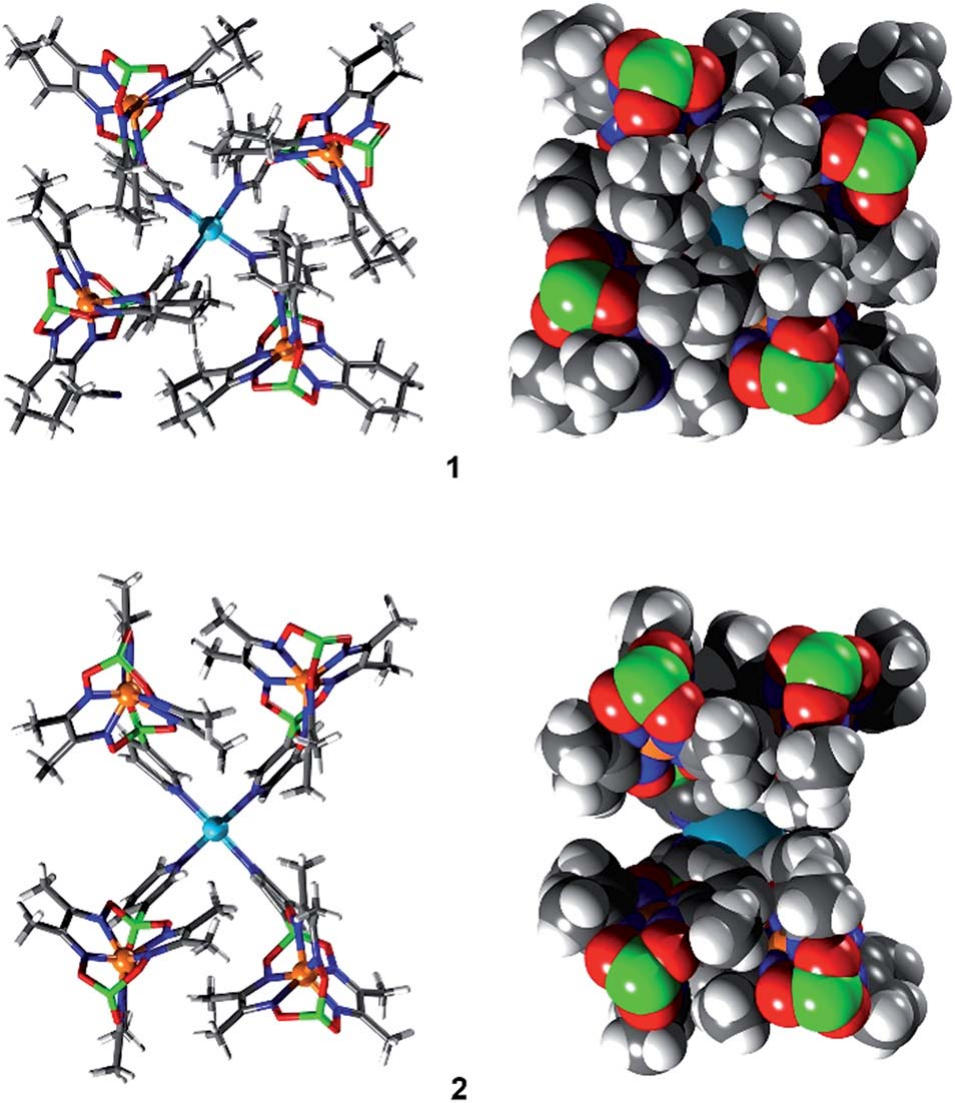
Parts of the structures of cage **1** (top) and cage **2** (bottom). The graphics depict the upper parts of the cages when viewed from the inside along the Pd···Pd axis, with wire representations on the left and space filling representations on the right. Solvent molecules and anions are omitted for clarity. Grey: C; dark blue: N; green: B; red: O; light blue: Pd, light grey: H and orange: Fe.

The structures of cages **1** and **3** show tight, interdigitated packing of the cyclohexyl side chains of the clathrochelate complexes (Fig. 3 and 4, top). The close packing is likely responsible for the hindered rotation observed by NMR spectroscopy. For cage **2**, which has smaller methyl side chains, no such tight packing is observed. We have previously shown that steric interactions between ligand side chains can thermodynamically destabilize a clathrochelate-based assembly^12*a*^ and, in view of these earlier results, we suspected that the sterically less congested cages **2** and **4** would prove to be more stable than the tightly packed cages **1** and **3**. Surprisingly, the opposite is true.

**Scheme 3:**
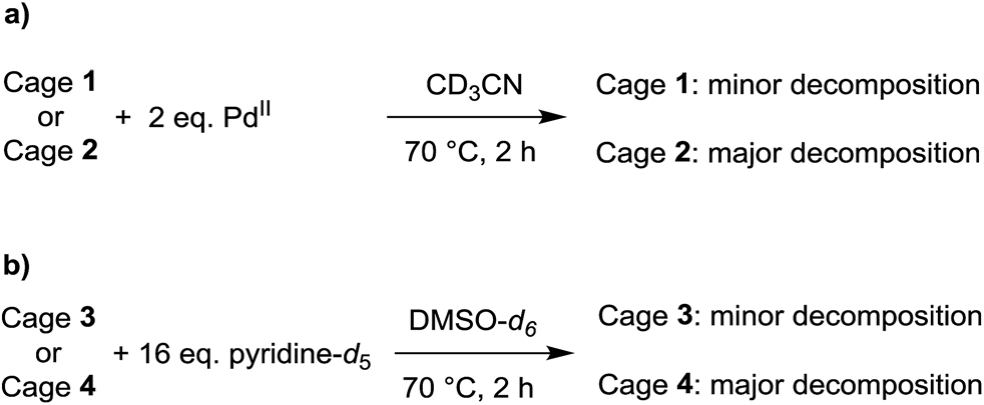
Decomposition experiments for evaluating the relative thermodynamic stability of the small cages **1** and **2**, as well as for the large cages **3** and **4**.

The relative thermodynamic stability of the smaller cages **1** and **2** in solution was examined by adding two equivalents of [Pd(CH_3_CN)_4_](BF_4_)_2_ to a solution of the cage in CD_3_CN (Scheme 3a). After heating to 70 °C for 2 h, the new equilibrium distribution was examined by ^1^H NMR spectroscopy. In the case of cage **1**, only minor decomposition was observed. In the case of cage **2**, on the other hand, substantial ligand rearrangement and cage decomposition was detected (ESI, Fig. S42 and S43†). These results indicate that cage **2** is less stable than cage **1** under these conditions. A stabilizing anionic template effect, resulting from the presence of a tightly encapsulated BF_4_^–^ anion in **1**, may offer an explanation for this trend.^20^

For the larger cages **3** and **4**, we used DMSO-*d*_6_ as a solvent due to solubility problems in CD_3_CN. Adding further equivalents of [Pd(CH_3_CN)_4_](BF_4_)_2_ to solutions of the cages did not result in detectable decomposition, presumably since Pd^2+^ ions are well solvated in DMSO and hence less efficient competition agents.

We therefore used an excess of pyridine-*d*_5_ to induce cage decomposition (Scheme 3b). The equilibrium composition was again determined by ^1^H NMR spectroscopy after a heating period of 2 h at 70 °C. Substantial decomposition was observed for cage **4**. In contrast, only low intensity new peaks were observed for cage **3**, indicating a superior stability of the latter (ESI, Fig. S44 and S45†). For the larger cages **3** and **4**, stabilizing effects of the BF_4_^–^ anions are unlikely to play an important role, as bound anions were observed neither by X-ray crystallography nor by NMR spectroscopy. Instead, we propose that the tightly packed cyclohexyl side chains are in fact a stabilizing factor for cage **3**. The packing of the lipophilic cyclohexyl groups buries a substantial amount of apolar surface area and, in a polar solvent such as DMSO, this clustering is expected to be energetically favorable.^21,22^

We have also attempted to examine the relative stabilities of the smaller cages **1** and **2** in DMSO. Interestingly, we were not able to achieve quantitative assembly of cage **2** from ligand **L2** and [Pd(CH_3_CN)_4_](BF_4_)_2_, despite the unproblematic formation of cage **1** in DMSO. Apparently, **2** is not sufficiently stable to allow the assembly process to occur in a strongly coordinating solvent such as DMSO. As in the cases of **3** and **4**, we propose that solvophobic effects contribute to the higher stability of cage **1** (cyclohexyl side chains) when compared to cage **2** (methyl side chains).

**Fig. 5:**
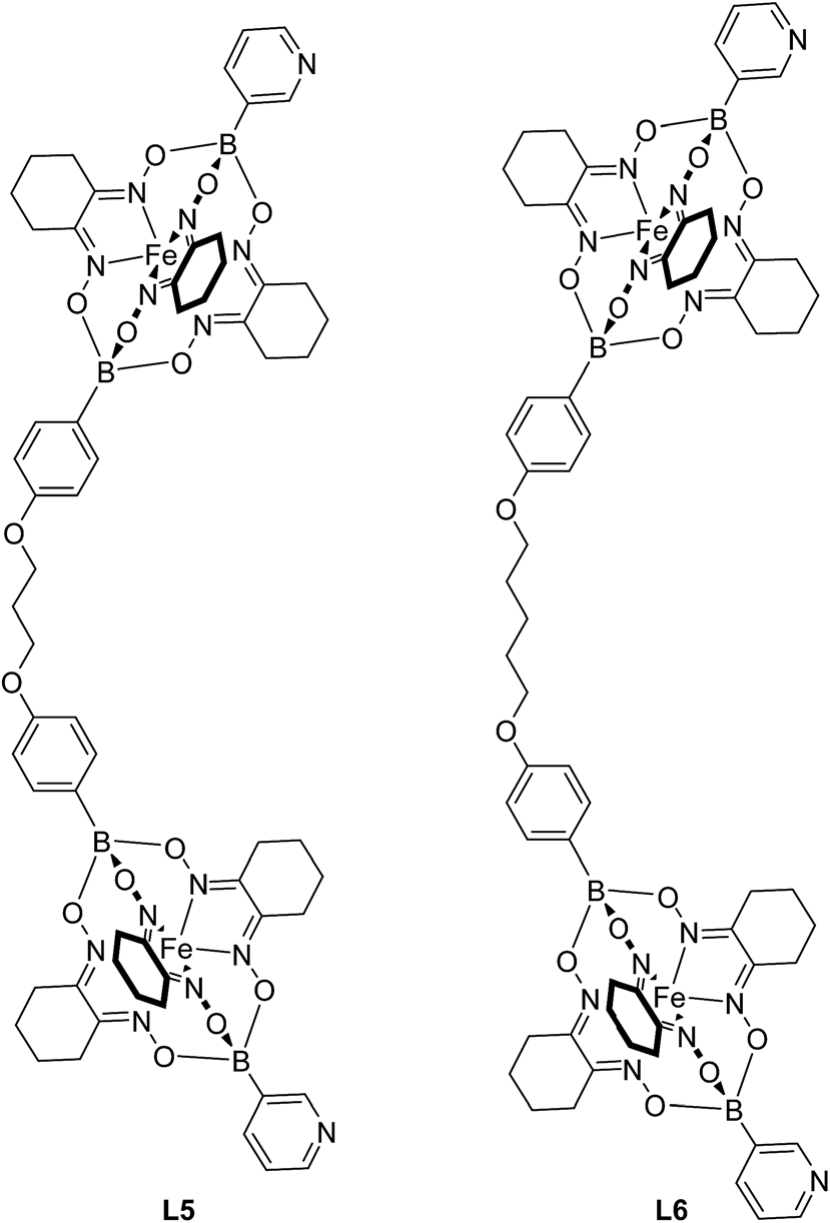
The metalloligands **L5** and **L6**.

The experiments described above suggest that clathrochelate-based metalloligands with cyclohexyl side chains are particularly suited for the construction of Pd^II^_2_L_4_ cages in polar solvents because solvophobic effects impart an additional stability to the assemblies. Therefore, we synthesized even larger cages using the extended metalloligands **L5** and **L6** (Fig. 5). The synthesis of these ligands was accomplished in a similar fashion as described for **L1** and **L3**, *i.e.* by using a mixture of 3-pyridylboronic acid and the corresponding diboronic acid along with nioxime and FeCl_2_. Purification by size exclusion chromatography gave the ligands **L5** and **L6** in yields of 48% and 49%, respectively. It should be noted that both ligands are rather flexible due to the presence of O(CH_2_)_*n*_O linkers (*n* = 3 or 5). In principle, highly flexible bipyridyl ligands can give rise to mononuclear complexes of the formula Pd^II^L_2_.^23^ For **L5** and **L6**, the sterically demanding clathrochelate complexes were expected to prevent such ‘back-biting’ of the ligand.

**Scheme 4:**
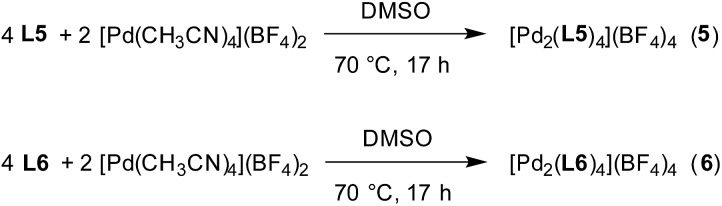
Synthesis of the cages **5** and **6**.

Finding a suitable solvent for reactions of **L5** and **L6** with [Pd(CH_3_CN)_4_](BF_4_)_2_ turned out to be challenging due to solubility problems. The best compromise was achieved with DMSO. The ligands **L5** and **L6** display very poor solubility in DMSO, but the addition of [Pd(CH_3_CN)_4_](BF_4_)_2_ resulted in the formation of a light orange solution along with some solid (Scheme 4). Analysis of the solution by high resolution mass spectrometry provided clear evidence for the presence of Pd^II^_2_L_4_ complexes (ESI, Fig. S40 and S41†). Unfortunately, the low concentrations of the complexes in solution precludes analysis by NMR.

**Fig. 6:**
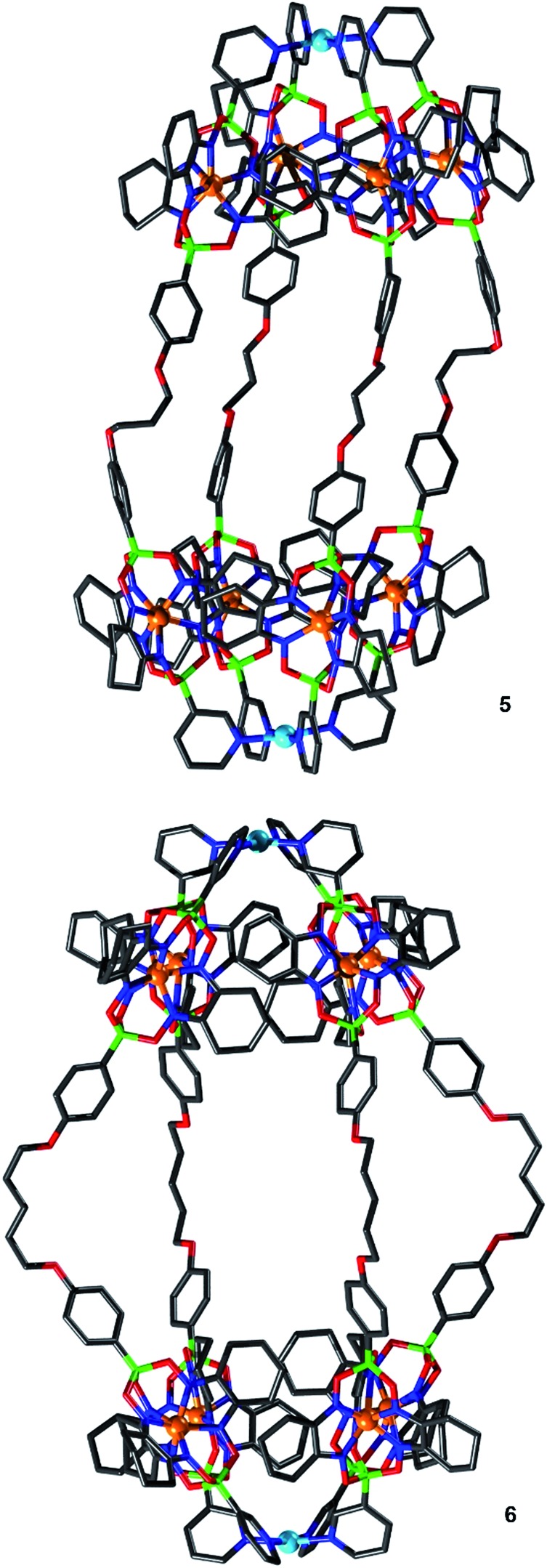
Molecular structures of the cages **5** (top) and **6** (bottom) as determined by single crystal X-ray diffraction. Hydrogen atoms, solvent molecules and anions are omitted for clarity. Grey: C; dark blue: N; green: B; red: O; light blue: Pd and orange: Fe.

Single crystals of **5** and **6** were obtained by slow diffusion of diisopropylether or diethyl ether into a solution of the cage in acetonitrile/DMSO (2 : 8). Crystallographic analyses show that Pd^II^_2_L_4_-type complexes had formed in both cases (Fig. 6). As observed for cages **1** and **3**, the assemblies feature tightly packed cyclohexyl side chains of the clathrochelate complexes adjacent to the palladium ions. The proximity of neighboring clathrochelate complexes is reflected by the average distance of the four adjacent Fe^II^ centers. The values observed for **5** (0.84 nm) and **6** (0.83 nm) are close to what was found for **1** (0.83 nm) and **3** (0.83 nm). Apparently, the presence of more flexible O(CH_2_)_*n*_O linkers in **5** and **6** does not result in a more relaxed arrangement of the clathrochelate complexes.

The O(CH_2_)_5_O linkers of cage **6** are bent outwards, whereas the O(CH_2_)_3_O groups of cage **5** are essentially straight. Consequently, the Pd···Pd distances of the two cages are similar (2.98 and 3.03 nm). The length of these cages is comparable to some of the longest cylindrical metallasupramolecular assemblies described to date.^24^

## Conclusions

We have synthesized and characterized novel, bent ditopic metalloligands with terminal 3-pyridyl groups. The preparation of these ligands is straightforward and relies on the Fe^II^-templated formation of clathrochelate complexes. In order to access long metalloligands with two pyridyl-capped clathrochelate complexes, we have used a mixture of a diboronic acid and 3-pyridylboronic acid. This statistical synthesis gives rise to a mixture of products. However, purification by size exclusion chromatography is unproblematic. The ligands were used for the assembly of cage structures of the general formula (Pd^II^_2_L_4_)(BF_4_)_4_. We characterized five of these assemblies by single crystal X-ray crystallography revealing remarkably large structures, spanning 2.0 nm to 3.0 nm. The thermodynamic stability of the cages was studied by competition experiments. These investigations revealed a surprising fact: cages based on ligands with sterically more demanding cyclohexyl side chains are more stable than cages based on ligands with smaller methyl side chains. We propose that the close packing of lipophilic cyclohexyl side chains contributes to the enhanced stability in polar solvents such as DMSO. Solvophobic effects of this kind are commonly observed for peptide or protein assemblies. For example, the aggregation of coiled-coil structures from amphiphilic α-helices is largely driven by the burial of hydrophobic surface area.^25^ However, in the area of metallasupramolecular chemistry, these effects are rarely used in a deliberate manner, and demonstrate exciting potential as a more general tool to stabilize large, hydrophobic assemblies in polar solvents.

## References

[cit1] Cook T. R., Stang P. J. (2015). Chem. Rev..

[cit2] Horner K. E., Miller M. A., Steed J. W., Sutcliffe P. M. (2016). Chem. Soc. Rev..

[cit3] Otte M. (2016). ACS Catal..

[cit4] Schmittel M. (2015). Chem. Commun..

[cit5] Obviously, one could also use a large number of large building blocks, but this approach would suffer from the drawbacks of option (a) and (b)

[cit6] Fujita D., Ueda Y., Sato S., Yokoyama H., Mizuno N., Kumasaka T., Fujita M. (2016). Chem.

[cit7] Engelhard D. M., Freye S., Grohe K., John M., Clever G. H. (2012). Angew. Chem., Int. Ed..

[cit8] Reiss P. S., Little M. A., Santolini V., Chong S. Y., Hasell T., Jelfs K. E., Briggs M. E., Cooper A. I. (2016). Chem.–Eur. J..

[cit9] Neelakandan P. P., Jiménez A., Thoburn J. D., Nitschke J. R. (2015). Angew. Chem., Int. Ed..

[cit10] Wise M. D., Ruggi A., Pascu M., Scopelliti R., Severin K. (2013). Chem. Sci..

[cit11] Li L., Fanna D. J., Shepherd N. D., Lindoy L. F., Li F. (2015). J. Inclusion Phenom. Macrocyclic Chem..

[cit12] Jansze S. M., Cecot G., Wise M. D., Zhurov K. O., Ronson T. K., Castilla A. M., Finelli A., Pattison P., Solari E., Scopelliti R., Zelinskii G. E., Volgzhanina A. V., Voloshin Y. Z., Nitschke J. R., Severin K. (2016). J. Am. Chem. Soc..

[cit13] Marmier M., Cecot G., Vologzhanina A. V., Bila J. L., Zivkovic I., Ronnow H. M., Nafradi B., Solari E., Pattison P., Scopelliti R., Severin K. (2016). Dalton Trans..

[cit14] (d) Voloshin Y. Z., Kostromina N. A. and Krämer R. K., Clathrochelates. Synthesis, Structure and Properties, Elsevier, Amsterdam, 2002.

[cit15] Cecot G., Alameddine B., Prior S., Zorzi R. D., Geremia S., Scopelliti R., Fadaei F. T., Solari E., Severin K. (2016). Chem. Commun..

[cit16] Löffler S., Lübben J., Wuttke A., Mata R. A., John M., Dittrich B., Clever G. H. (2016). Chem. Sci..

[cit17] Frank M., Johnstone M. D., Clever G. H. (2016). Chem.–Eur. J..

[cit18] Li Y.-H., Jiang J.-J., Fan Y.-Z., Wei Z.-W., Chen C.-X., Yu H.-J., Zheng S.-P., Fenske D., Su C.-Y., Barboiu M. (2016). Chem. Commun..

[cit19] Kaiser F., Schmidt A., Heydenreuter W., Altmann P. J., Casini A., Sieber S. A., Kühn F. E. (2016). Eur. J. Inorg. Chem..

[cit20] Custelcean R. (2014). Chem. Soc. Rev..

[cit21] Yang L., Adam C., Cockroft S. L. (2015). J. Am. Chem. Soc..

[cit22] For the kinetic stabilization of Pd^II^ _2_L_4_-type cages by intramolecular hydrogen bonding see: Preston D. McNeill S. M. Lewis J. E. M. Giles G. I. Crowley J. D., Dalton Trans., 2016, 45 , 8050 . 27074828 10.1039/c6dt00133e

[cit23] Yue N. L. S., Jennings M. C., Puddephatt R. J. (2016). Inorg. Chim. Acta.

[cit24] Wood C. S., Ronson T. K., McConnell A. J., Roberts D. A., Nitschke J. R. (2016). Chem. Sci..

[cit25] Luopas A. N., Gruber M. (2005). Adv. Protein Chem..

