# Supplementary material for: PdII 2L4-type coordination cages up to three nanometers in size
Source: Chem Sci. 2016 Nov 18;8(3):1901–8. doi: 10.1039/c6sc04732g (PMC5444114; doi:10.1039/c6sc04732g)
Supplement: Supplementary file 1 [file SC-008-C6SC04732G-s001.pdf]

Electronic Supplementary Material (ESI) for Chemical Science.
This journal is © The Royal Society of Chemistry 2016

Electronic Supplementary Information

$Pd^{II}_2L_4$-type coordination cages up to three nanometers in size

Suzanne Jansze,[a] Matthew D. Wise,[a] Anna V. Vologzhanina,[b] Rosario Scopelliti,[a] and Kay Severin[a]

[a] Institut des Sciences et Ingénierie Chimiques, Ecole Polytechnique Fédérale de Lausanne (EPFL), 1015 Lausanne, Switzerland

[b] Nesmeyanov Institute of Organoelement Compounds of the Russian Academy of Sciences, 119991 Moscow, Russia

Table of Contents

1. General S2
2. Synthetic procedures S3
3. NMR spectra S8
4. Mass spectra Pd_2L_4 coordination cages S26
5. Destruction experiments S32
6. Single crystal X-ray analysis S36
7. References S40

1. General

All chemicals were obtained from commercial sources (see below) and used without further purification unless stated otherwise. Bis(bromophenyl)methane (**A**) was synthesized following a literature procedure.[1] Solvents were dried using a solvent purification system from Innovative Technologies, Inc.. Reactions were carried out under an atmosphere of dry N_2 using standard Schlenk techniques.

NMR spectra were obtained on a Bruker DRX (^{1}H: 400 MHz, ^{13}C: 100 MHz) equipped with a BBO 5 mm probe and a Bruker Avance III spectrometer (^{1}H: 400 MHz) equipped with a 5 mm BBFO-Plus probe.

The chemical shifts are reported in parts per million δ (ppm) referenced to the residual solvent signal. All spectra were recorded at 298 K, unless stated otherwise. The analysis of NMR spectra was performed with MestreNova and for the DOSY analysis the Baysian DOSY transform from MestreNova was used.

Routine ESI-MS data were acquired on a Q-TOF Ultima mass spectrometer (Waters) operated in the positive ionization mode and fitted with a standard Z-spray ion source equipped with the Lock-Spray interface. Data were processed using the MassLynx 4.1 software.

High resolution mass spectra were acquired for pure, pre-synthesized samples of all cages. The analytes were diluted in acetonitrile to a final concentration of ~10-20 μM. High resolution mass spectrometry experiments were carried out using a hybrid ion trap-Orbitrap Fourier transform mass spectrometer, Orbitrap Elite (Thermo Scientific) equipped with a TriVersa Nanomate (Advion) nano-electrospray ionization source. Mass spectra were acquired with a minimum resolution setting of 120,000 at 400 m/z. To reduce the degree of analyte gas phase reactions leading to side products unrelated to solution phase, the transfer capillary temperature was lowered to 50 °C. Experimental parameters were controlled via standard and advanced data acquisition software. Post-acquisition analysis was performed using vendor software, Xcalibur (Thermo Scientific), and ChemCalc (http://www.chemcalc.org/) web tool.[2]

Commercial sources:

1,3-Dibromopropane – AlfaAesar

1,3-Phenylenediboronic acid – FluoroChem

1,4-Dibromobenzene - VWR International SA

1,5 Dibromopentane – TCI

4-Bromobenzaldehyde – Maybridge

4-Bromophenol – Sigma Aldrich

Dimethylglyoxime – Apollo Scientific

Iron(II)chloride anhydrous – VWR International SA

Nioxime – TCI

p-Tolylboronic acid – Sigma Aldrich

Pyridine-3-boronic acid – FluoroChem

Tetrakis(acetonitrile)palladium(II) tetrafluoroborate – ABCR

2. Synthetic procedures

2.1 Synthesis of methylenebis(1,4-phenylene)diboronic acid (B)

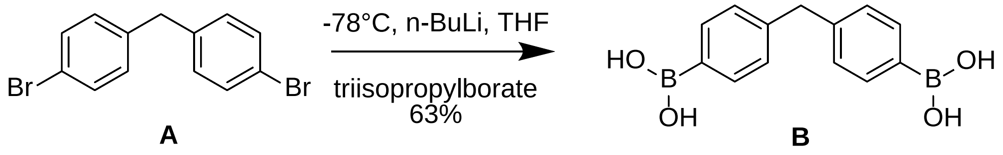

Scheme S1: Synthesis of diboronic **B** from bis(4-bromophenyl)methane (**A**).

A solution of bis(4-bromophenyl)methane (**A**) (3.0 g, 9.2 mmol) in THF (40 mL) was cooled to −78°C. N-butyllithium in hexane (2.5 M, 8.1 mL, 20.2 mmol, 2.2 eq.) was slowly added and stirred for an additional 30 min before triisopropylborate (3.8 g, 4.7 mL, 20.2 mmol, 2.2 eq.) was added. The reaction mixture was then left to warm up to r.t. overnight. Aqueous HCl (1 M, 20 mL) was added to quench the reaction and the solvent was removed under reduced pressure. A solid was collected, which was washed with water (3 x 50 mL) and with a 1:1 pentane/DCM mixture (2 x 50 mL) and dried by air. Diboronic acid **B** was obtained in the form of a white powder (1.9 g, 5.8 mmol, 63%).

^{1}H NMR (400 MHz, DMSO-d_6) δ 7.91 (s, 4H), 7.66 (d, J = 7.5 Hz, 4H), 7.14 (d, J = 7.6 Hz, 4H), 3.89 (s, 2H). ^{13}C NMR (101 MHz, DMSO-d_6) δ 143.06, 134.31, 127.81, 40.15, (C-B not detected). HRMS (ESI): *m/z* calculated for $C_{17}H_{22}B_2NaO_4$ [M+4 CH_2+Na]$^+$ (4 x methoxy adduct, from methanol as solvent) 335.1602, found 335.1609.

2.2 Synthesis of ((propane-1,3-diylbis(oxy))bis(4,1-phenylene)diboronic acid (D)

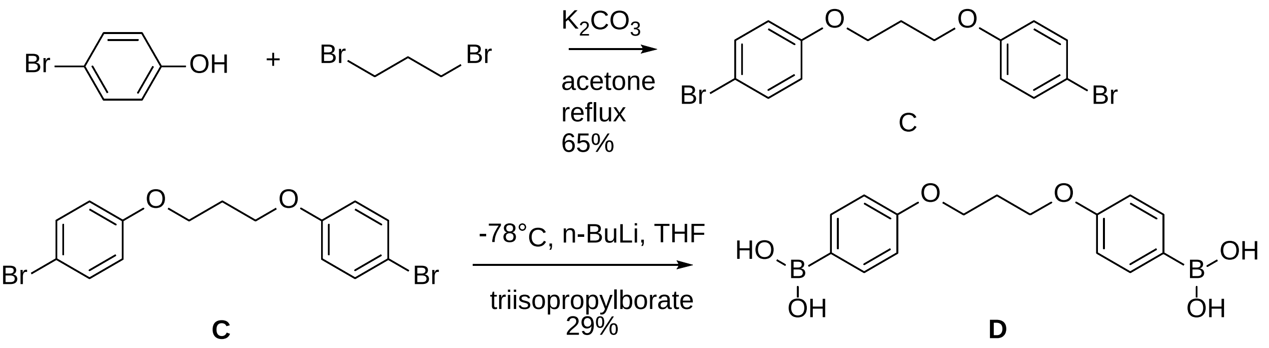

Scheme S2: Synthesis of diboronic acid **D**.

4-Bromophenol (10 g, 57.8 mmol, 2 eq.), 1,5-dibromopane (5.8 g, 28.9 mmol 1 eq.) and K_2CO_3 (60 g, 780 mmol 7.5 eq.) were added to acetone (250 mL) and the mixture was heated under reflux overnight. The reaction mixture was cooled to r.t. and the white solid was filtered and washed with acetone (200 mL) and DCM (100 mL). The organic layer was evaporated under reduced pressure to obtain the dibromo compound **C** as a white powder (7.2 g, 18.7 mmol, 65%).

^{1}H NMR (400 MHz, CD_2Cl_2) δ 7.37 (d, J = 8.9 Hz, 4H), 6.81 (d, J = 8.9 Hz, 4H), 4.11 (t, J = 6.1 Hz, 4H), 2.23 (p, J = 6.1 Hz, 2H). ^{13}C NMR (101 MHz, CD_2Cl_2) δ 158.66, 132.76, 116.88, 113.22, 65.21, 29.67. HRMS (APCI): *m/z* calculated for $C_{15}H_{15}Br_2O_2$ [M+H]$^+$ 385.9341, found 385.9327.

A solution of the dibromo starting material **C** (5.0 g, 13.0 mmol) in THF (40 mL) was cooled to −78°C. N-butyllithium in hexane (2.5 M, 11.4 mL, 28.59 mmol, 2.2 eq.) was slowly added and stirred for an additional 30 min before triisopropylborate (5.4 g, 6.6 mL, 28.5 mmol, 2.2 eq.) was added. The reaction mixture was then left to warm up to r.t. overnight. Aqueous HCl (1 M, 10 mL) was added to quench the

reaction and the solvent was removed under reduced pressure. A solid was collected, which was washed with water (3 x 50 mL) and with a 1:1 DCM/MeOH mixture (2 x 50 mL) and dried by air. The diboronic acid **D** was obtained in the form of a white powder (1.2 g, 3.8 mmol, 29%).

^{1}H NMR (400 MHz, DMSO-d_6) δ 7.83 (s, 4H), 7.73 (d, J = 8.3 Hz, 4H), 6.91 (d, J = 8.4 Hz, 4H), 4.15 (t, J = 6.1 Hz, 4H), 2.18 (p, J = 5.9 Hz, 2H). ^{13}C NMR (101 MHz, DMSO-d_6) δ 160.15, 135.85, 113.42, 63.93, 28.63, (C-B not detected). HRMS (ESI): *m/z* calculated for $C_{19}H_{26}B_2NaO_6$ [M+4 CH_2+Na]$^+$ (4 x methoxy adduct, from methanol solvent) 395.1813, found 395.1811.

2.3 Synthesis of ((pentane-1,5-diylbis(oxy))bis(4,1-phenylene)diboronic acid (F)

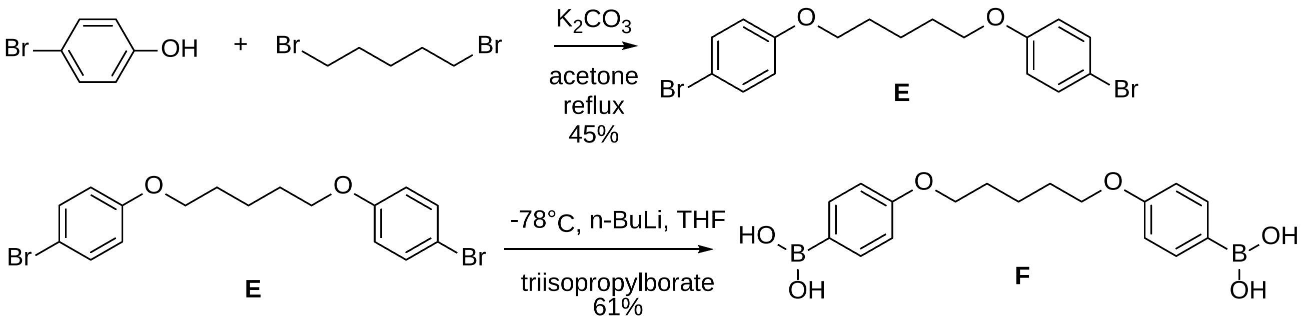

Scheme S3: Synthesis of diboronic acid **F**.

4-Bromophenol (10 g, 57.8 mmol, 2 eq.), 1,5-dibromopentane (6.7 g, 28.9 mmol 1 eq.) and K_2CO_3 (60 g, 780 mmol 7.5 eq.) were added to acetone (250 mL) and the mixture was heated under reflux overnight. The reaction mixture was cooled to r.t. and the white solid was filtered of and washed with acetone (200 mL). The organic layer was evaporated under reduced pressure and the remaining solid was washed with hexane (50 mL) and dried to obtain the dibromo compound **E** as a white powder (5.1 g, 12.4 mmol, 45%).

^{1}H NMR (400 MHz, CD_2Cl_2) δ 7.37 (d, J = 8.9 Hz, 4H), 6.79 (d, J = 8.9 Hz, 4H), 3.95 (t, J = 6.4 Hz, 4H), 1.83 (p, J = 6.6 Hz, 4H), 1.62 (p, J = 7.7, 7.1 Hz, 2H). ^{13}C NMR (101 MHz, CD_2Cl_2) δ 158.87, 132.71, 116.86, 112.96, 68.63, 29.46, 23.16. HRMS (APCI): *m/z* calculated for $C_{17}H_{19}Br_2O_2$ [M+H]$^+$ 414.9732, found 414.9724

A solution of the dibromo starting material **E** (3.0 g, 7.2 mmol) in THF (40 mL) was cooled to −78°C. N-butyllithium in hexane (2.5 M, 6.4 mL, 15.9 mmol, 2.2 eq.) was slowly added and stirred for an additional 30 min before triisopropylborate (3.0 g, 3.7 mL, 15.9 mmol, 2.2 eq.) was added. The reaction mixture was then left to warm up to r.t. overnight. Aqueous HCl (1 M, 20 mL) was added to quench the reaction and the solvent was removed under reduced pressure. A solid was collected, which is washed with water (3 x 50 mL) and with a 1:1 pentane/DCM mixture (2 x 50 mL) and dried by air. The diboronic acid **F** was obtained in the form of a white powder (1.8 g, 4.3 mmol, 61%).

^{1}H NMR (400 MHz, DMSO-d_6) δ 7.82 (s, 4H), 7.70 (d, J = 7.5 Hz, 4H), 6.86 (d, J = 7.5 Hz, 4H), 3.98 (t, J = 6.0 Hz, 4H), 1.86 – 1.69 (m, 4H), 1.69 – 1.40 (m, 2H). ^{13}C NMR (101 MHz, DMSO-d_6) δ 174.83, 150.31, 127.88, 81.57, 42.93, 36.76, (C-B not detected). HRMS (ESI): *m/z* calculated for $C_{21}H_{30}B_2NaO_6$ [M+4 CH_2+Na]$^+$ (4 x methoxy adduct, from methanol solvent) 423.2126, found 423.2140.

2.3 Synthesis of double clathrochelate (L1–L6)

General procedure for the synthesis of double clathrochelate (L1–L6)

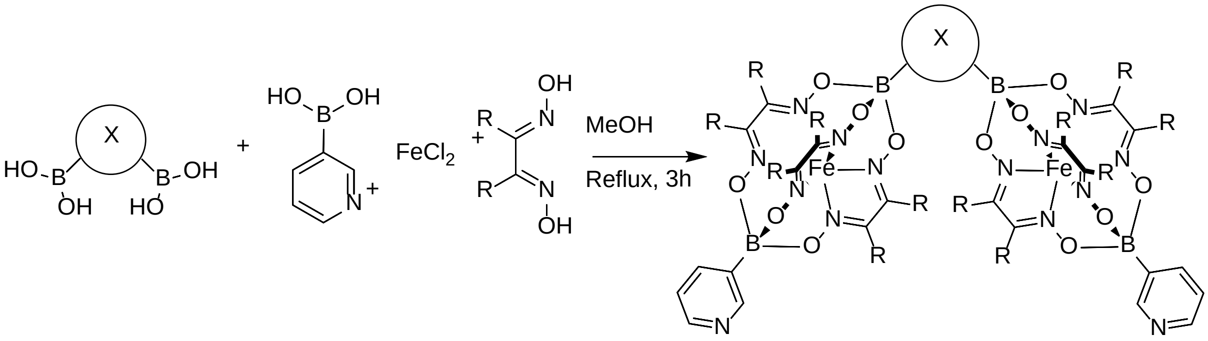

Scheme S4. Synthesis of double clathrochelate **L1–L6**

Anhydrous $FeCl_2$ (4 eq.) and the respective dioxime (12 eq.) were dissolved in MeOH (15 mL). In a separate flask, the respective diboronic acid (100 mg, 1 eq.) and 3-pyridine boronic acid (6 eq.) were dissolved in methanol (130 mL), acetone (5 mL), and water (2 mL) and heated to reflux and stirred for 30 min. The pre-prepared mixture of dioxime and $FeCl_2$, was added to the boronic acid mixture, the mixture was heated to reflux for an additional 2 h, before the solvent was removed under reduced pressure. The remaining solid was dissolved in $CHCl_3$ (100 mL), filtered and washed with a saturated aqueous solution of sodium EDTA and 5% ammonia (100 mL). The organic phase was dried over $MgSO_4$, and evaporated under reduced pressure. The solid was pre-purified by a short silica column (150 g silica, 10% MeOH in DCM) to remove any polymeric material. The dark red fractions were evaporated under reduced pressure, the solid was dissolved in DCM (10 mL), filtered over H-PTFE 20/25 syringe filters and separated on a size exclusion column (200 g, dry weight, Bio-Beads S-X3 in DCM). The pure fractions (checked by MS, pos. mode), were combined and washed with saturated $NaHCO_3$ solution, dried over $MgSO_4$ and the solvent was removed under reduced pressure to yield a red powder as the double clathrochelate.

Table S1: Amounts used for the synthesis of double clathrochelates **L1–L6**. BA is boronic acid, CC is clathrochelate.

**Double CC**	**Di-BA**	**4 eq. FeCl$_2$**		**12 eq. nioxime**		**1 eq. Di BA**		**6 eq. 3-pyridineBA**		**Yield double clathrochelate**		
**#**	**#**	**mg**	**mmol**	**mg**	**mmol**	**mg**	**mmol**	**mg**	**µmol**	**mg**	**mmol**	**%**
**L1**	**-**	306	2.4	1029	7.2	100	0.60	445	3.6	435	0.35	59
**L3**	**B**	198	1.6	667	4.7	100	0.39	288	2.3	262	0.20	51

**Double CC**	**Di-BA**	**4 eq. FeCl$_2$**		**12 eq. dimethyl-glyoxime**		**1 eq. Di BA**		**6 eq. 3-pyridineBA**		**Yield double clathrochelate**		
**#**	**#**	**mg**	**mmol**	**mg**	**mmol**	**mg**	**mmol**	**mg**	**µmol**	**mg**	**mmol**	**%**
**L2**	**-**	306	2.4	841	7.2	100	0.60	445	3.6	264	0.25	41
**L4**	**B**	198	1.6	545	4.7	100	0.39	288	2.3	177	0.15	39

**Double CC**	**Di-BA**	**4 eq. FeCl$_2$**		**12 eq. nioxime**		**1 eq. Di BA**		**6 eq. 3-pyridineBA**		**Yield double clathrochelate**		
**#**	**#**	**mg**	**mmol**	**mg**	**mmol**	**mg**	**mmol**	**mg**	**µmol**	**mg**	**mmol**	**%**
**L5**	**D**	160	1.3	540	3.8	100	0.32	233	1.9	326	0.24	48
**L6**	**F**	147	1.2	496	3.5	100	0.29	214	1.7	200	0.14	49

Characterization for double clathrochelate L1–L6

L1: ^{1}H NMR (400 MHz, CD_2Cl_2) δ 8.72 (s, 2H), 8.43 (d, J = 3.5 Hz, 2H), 7.88 (d, J = 6.4 Hz, 3H), 7.51 (d, J = 7.3 Hz, 2H), 7.21 - 7.16 (m, 3H), 2.85 (d, J = 13.4 Hz, 24H), 1.74 (s, 24H). ^{13}C NMR (101 MHz, CD_2Cl_2) δ 152.98, 152.74, 152.32, 148.90, 140.22, 135.74, 131.72, 126.81, 123.50, 26.81, 26.75, 22.20, (C-B not detected). HRMS (ESI): *m/z* calculated for $C_{52}H_{62}B_4Fe_2N_{14}O_{12}$ $[M+2H]^{2+}$ 615.1888, found 615.1895.

L2: ^{1}H NMR CD_2Cl_2) δ 8.91 (s, 2H), 8.58 (broad d, 2H), 8.11 (s, 1H), 8.03 (d, J = 7.3 Hz, 2H), 7.72 (d, J = 7.3 Hz, 2H), 7.38 (t, J = 7.3 Hz, 1H), 7.36 – 7.27 (m, 2H), 2.50 (s, 18H), 2.47 (s, 18H). ^{13}C NMR ^{13}C NMR (101 MHz, CD_2Cl_2) δ 152.92, 152.41, 152.00, 148.82, 139.19, 135.04, 131.25, 126.31, 122.81, 13.10, (C-B not detected). HRMS (ESI): *m/z* calculated for $C_{40}H_{50}B_4Fe_2N_{14}O_{12}$ $[M+2H]^{2+}$ 537.1407, found 537.1396.

Single crystals of sufficient quality for X-ray analysis were obtained for the double clathrochelates **L1** and **L2** by slow diffusion of diethyl ether into a solution of the compounds in DCM. See the last chapter of this SI for more details.

L3: ^{1}H NMR (400 MHz, CD_2Cl_2) δ 8.80 (s, 2H), 8.50 (broad d, 2H), 7.92 (d, J = 7.1 Hz, 2H), 7.57 (d, J = 7.5 Hz, 4H), 7.25 – 7.17 (m, 6H), 3.98 (s, 2H), 2.91 (s, 24H), 1.81 (s, 24H). ^{13}C NMR (101 MHz, CD_2Cl_2) δ 153.48, 152.74, 152.46, 149.41, 141.77, 139.75, 132.29, 128.45, 123.34, 42.61, 26.77, 22.16, (C-B not detected). HRMS (ESI): *m/z* calculated for $C_{59}H_{68}B_4Fe_2N_{14}O_{12}$ $[M+2H]^{2+}$ 660.2124, found 660.2134.

L4: ^{1}H NMR (400 MHz, CD_2Cl_2) δ 8.89 (s, 2H), 8.57 (broad d, 2H), 8.02 (d, J = 6.5 Hz, 2H), 7.67 (d, J = 6.9 Hz, 4H), 7.38 – 7.05 (m, 6H), 4.05 (s, 2H), 2.46 (s, 36H). ^{13}C NMR (101 MHz, CD_2Cl_2) δ 152.92, 152.43, 152.15, 148.84, 141.23, 139.19, 131.76, 127.87, 122.79, 42.07, 13.13, 13.10, (C-B not detected). HRMS (ESI): *m/z* calculated for $C_{47}H_{56}B_4Fe_2N_{14}O_{12}$ $[M+2H]^{2+}$ 582.1640, found 582.1631.

L5: ^{1}H NMR (400 MHz, CD_2Cl_2) δ 8.81 (s, 2H), 8.51 (broad d, 2H), 7.97 (d, J = 7.1 Hz, 2H), 7.57 (d, J = 8.1 Hz, 4H), 7.27 (t, J = 8.0 Hz, 2H), 6.89 (d, J = 8.1 Hz, 4H), 4.18 (t, J = 5.9 Hz, 4H), 2.91 (s, 24H), 2.27 (t, J = 8.0 Hz 2H), 1.81 (s, 24H). ^{13}C NMR (101 MHz, CD_2Cl_2) δ 159.48, 152.85, 152.77, 152.41, 148.78, 140.34, 133.40, 123.53, 114.11, 65.02, 30.04, 26.78, 22.15, (C-B not detected). HRMS (ESI): *m/z* calculated for $C_{61}H_{72}B_4Fe_2N_{14}O_{14}$ $[M+2H]^{2+}$ 690.2230, found 690.2236.

L6: ^{1}H NMR (400 MHz, CD_2Cl_2) δ 8.80 (s, 2H), 8.51 (s, 2H), 7.93 (d, J = 6.1 Hz, 2H), 7.56 (d, J = 7.3 Hz, 4H), 7.24 (s, 2H), 6.87 (d, J = 6.9 Hz, 4H), 4.02 (broad t, 4H), 2.91 (s, 24H), 1.92-1.75 (m, 28H), 1.68 (broad t, 2H). ^{13}C NMR (101 MHz, CD_2Cl_2) δ 159.66, 153.35, 152.40, 149.29, 139.88, 133.37, 123.38, 114.09, 68.18, 29.78, 26.78, 23.33, 22.16, (C-B not detected). HRMS (ESI): *m/z* calculated for $C_{63}H_{76}B_4Fe_2N_{14}O_{14}$ $[M+2H]^{2+}$ 704.2387, found 704.2383.

2.4 General synthesis procedure for Pd_2L_4 coordination cages.

To the double clahtrochelate ligand (see Table S2 for amounts, 2.2 µmol, 2 eq.) and $[Pd(CH_3CN)_4](BF_4)_2$ (0.5 mg, 1.1 µmol, 1 eq.) 0.6 mL of solvent (CD_3CN or DMSO-d_6) was added. The solution was heated at 70 °C for 17 h, in which the solution went from turbid to a clear red solution with everything dissolved. NMR shows full conversion to yield the Pd_2L_4 coordination cages. (except in the cases of double clatrhochelate (**5**) and (**6**) where there was a small amount of precipitate).

Table S2: The amounts of the double clathrochelates used for the synthesis of the M_2L_4 coordination cages.

Double clathrochelate #	Amount used (mg)
**L1**	2.8
**L2**	2.4
**L3**	3.0
**L4**	2.6
**L5**	3.1
**L6**	3.2

For characterization data see below.

3. NMR spectra

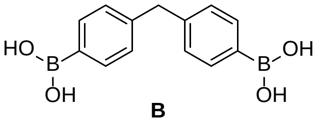

B

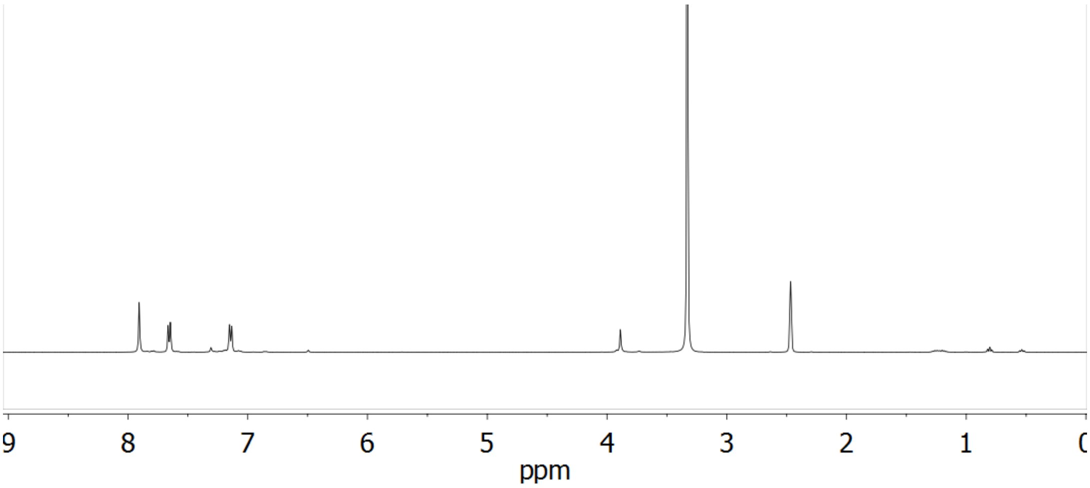

Figure S1. ^{1}H NMR spectrum of the diboronic acid **B** in DMSO-d_6.

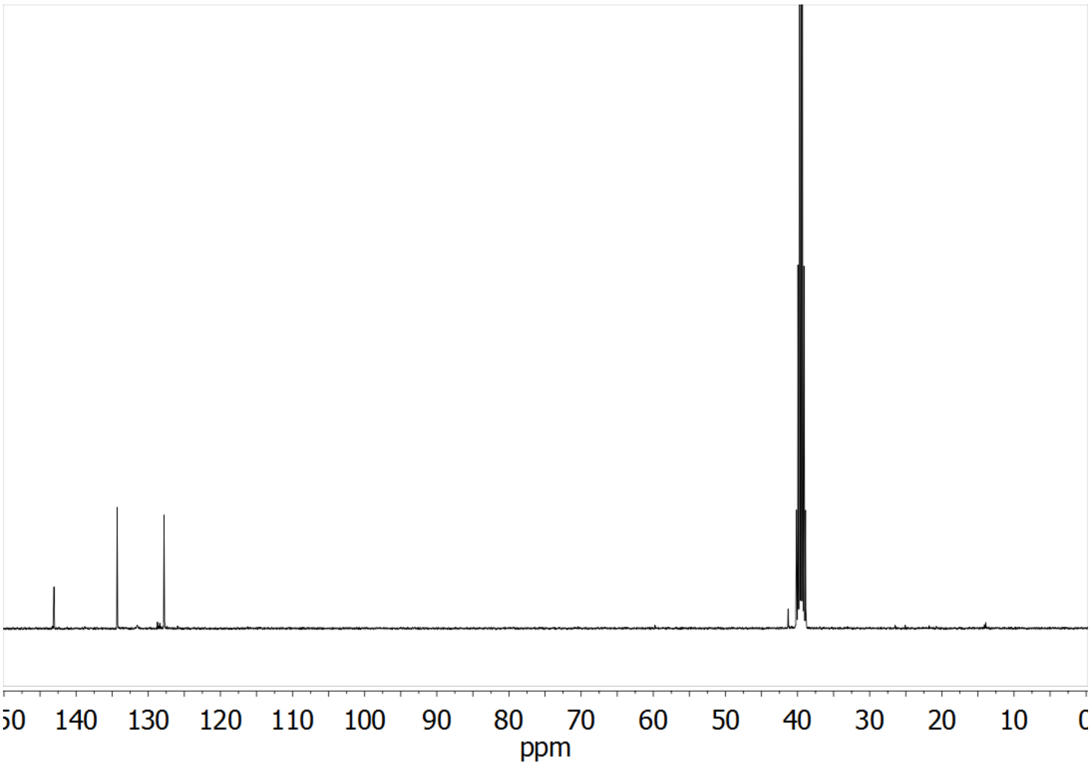

Figure S2. ^{13}C NMR spectrum of the diboronic acid **B** in DMSO-d_6.

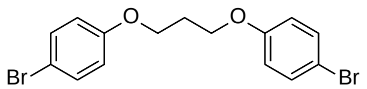

C

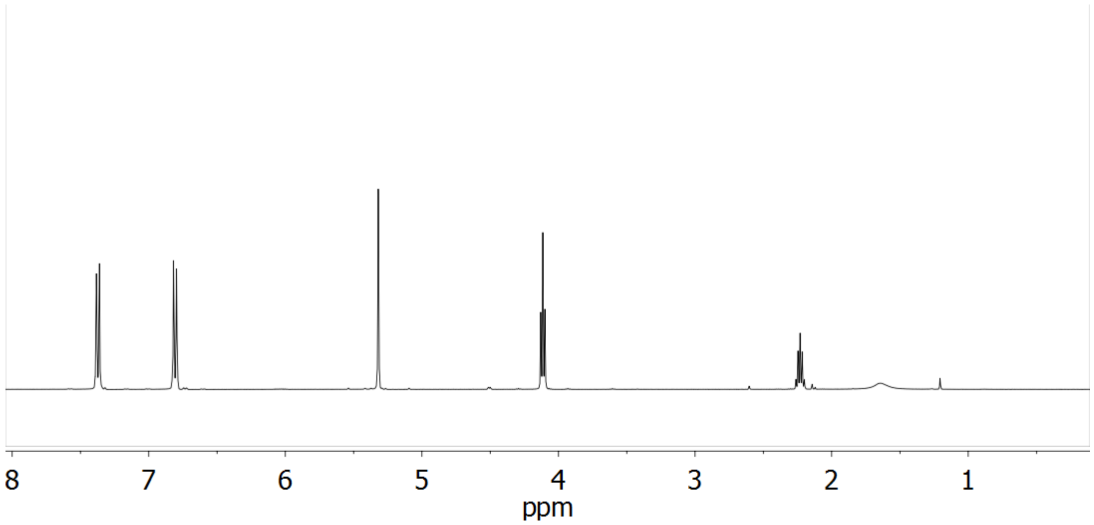

Figure S3. ^{1}H NMR spectrum of the dibromo compound **C** in CD_2Cl_2.

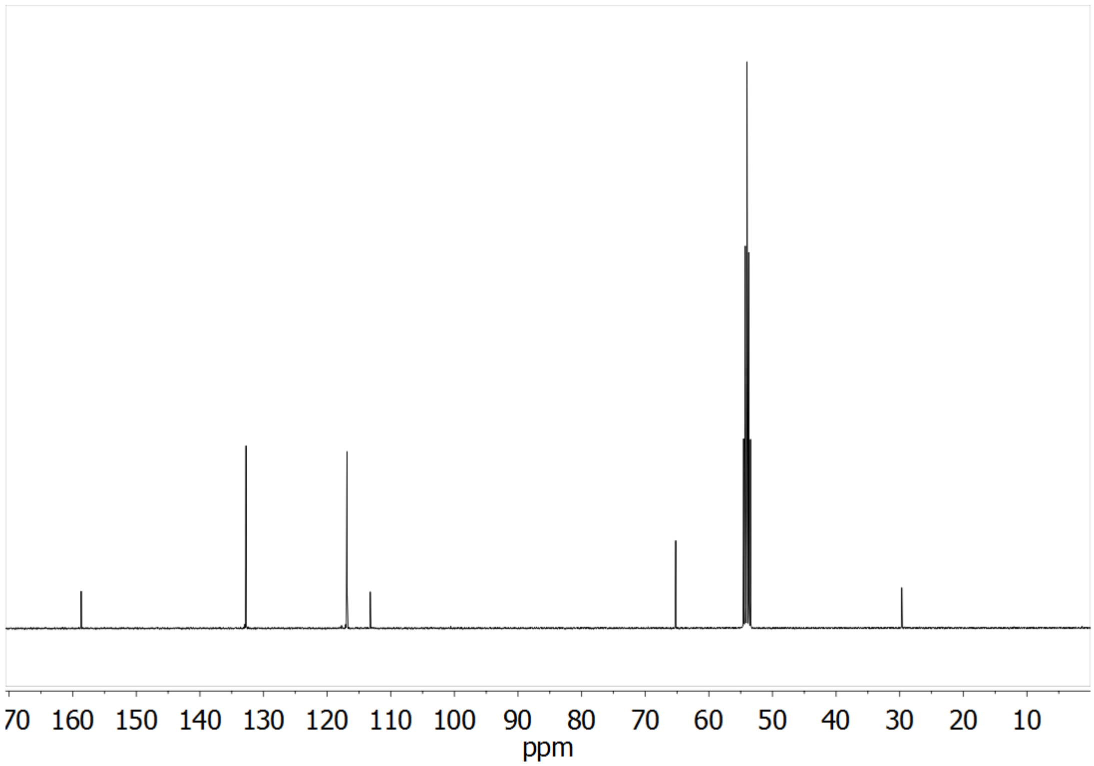

Figure S4. ^{13}C NMR spectrum of the dibromo compound **C** in CD_2Cl_2.

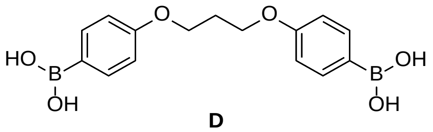

D

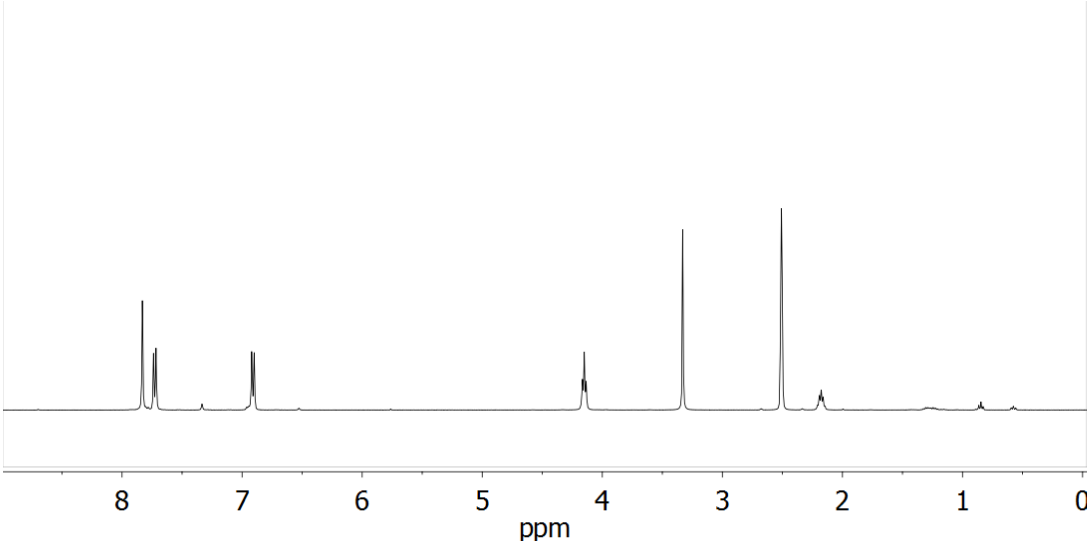

Figure S5. ^{1}H NMR spectrum of the diboronic acid **D** in DMSO-d_6.

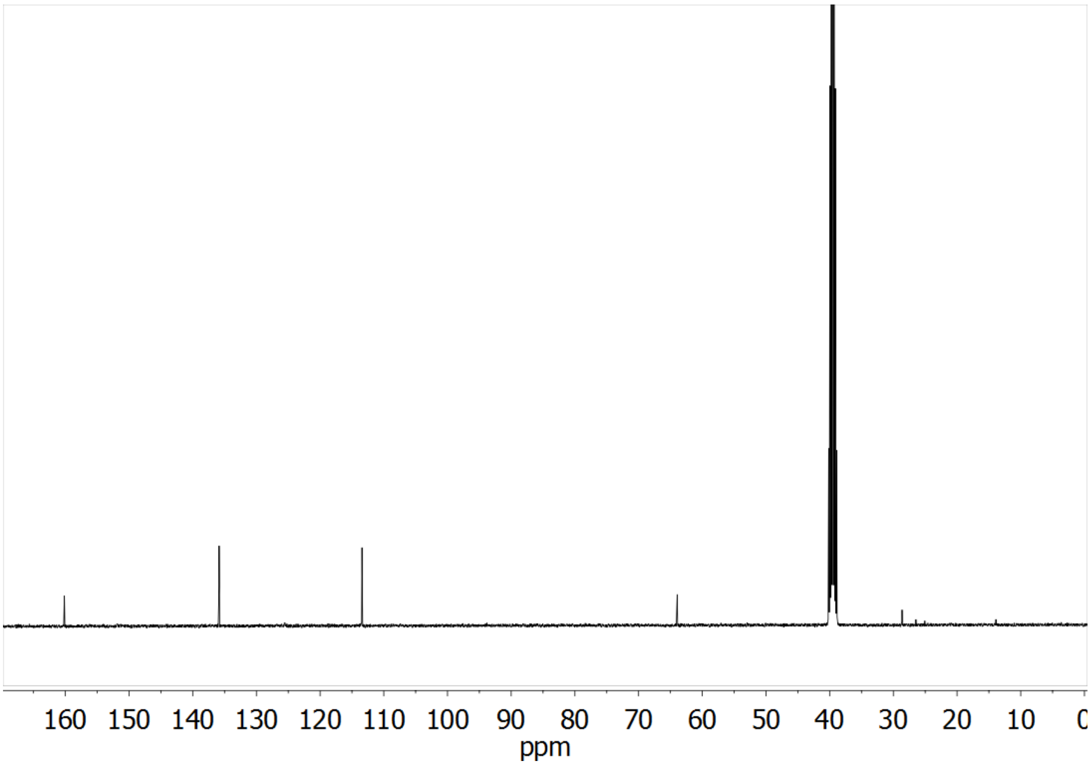

Figure S6. ^{13}C NMR spectrum of the diboronic acid **D** in DMSO-d_6.

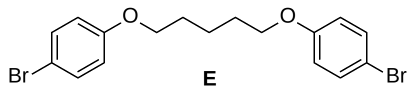

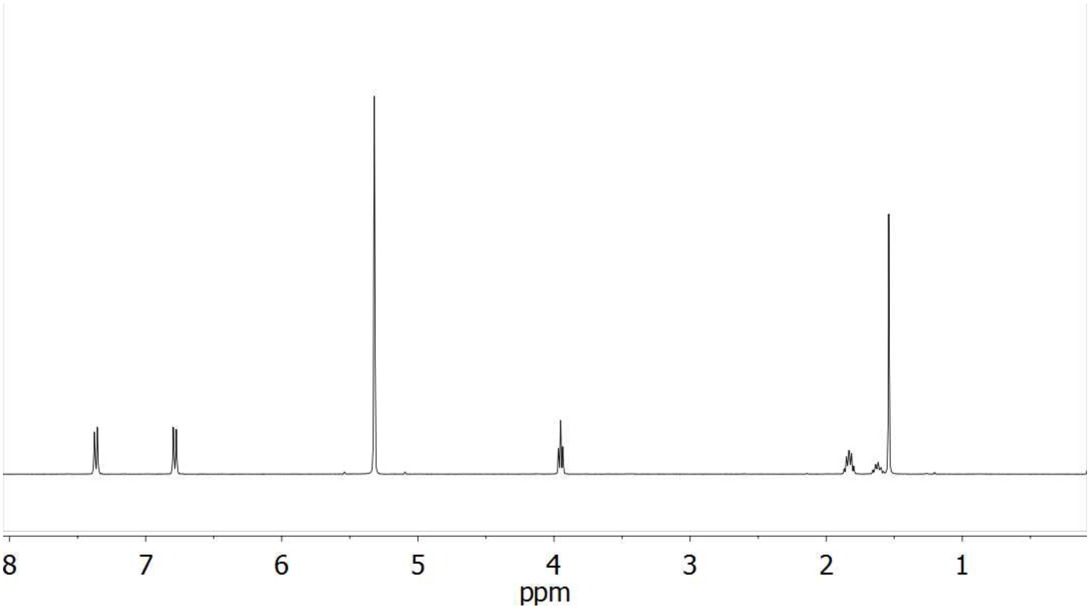

Figure S7. ^{1}H NMR spectrum of dibromo compound **E** in CD_2Cl_2.

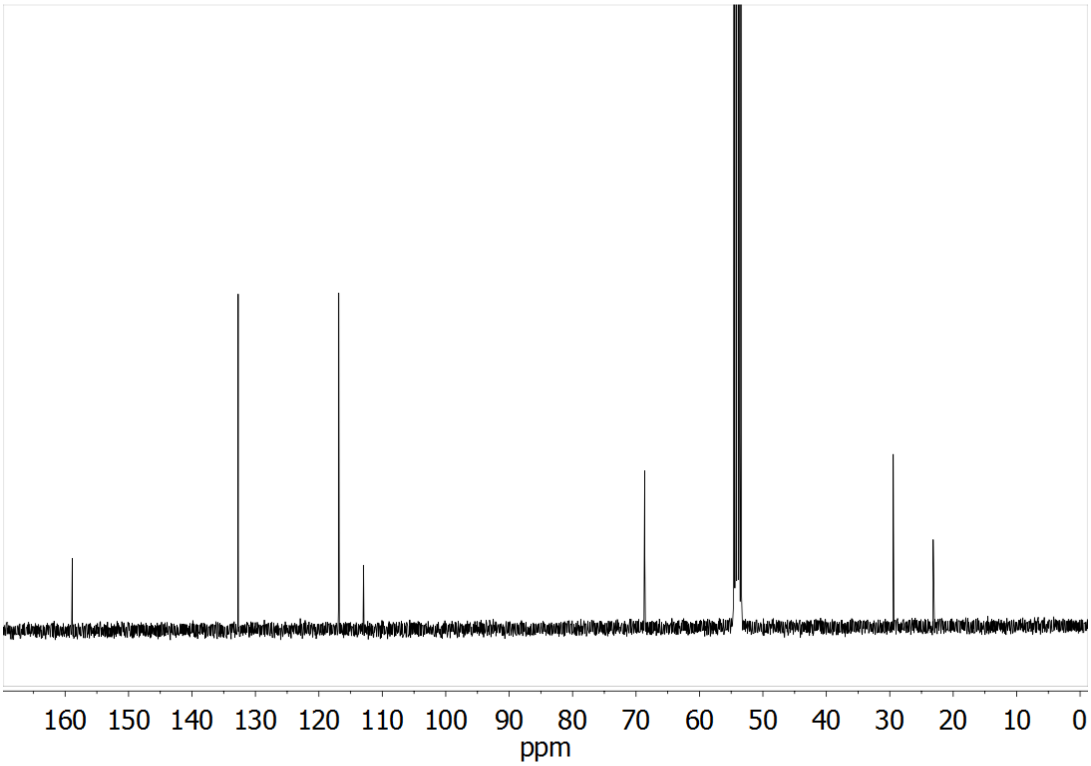

Figure S8. ^{13}C NMR spectrum of dibromo compound **E** in CD_2Cl_2.

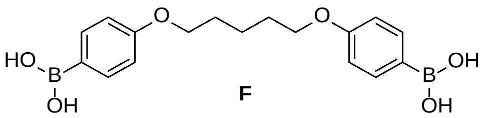

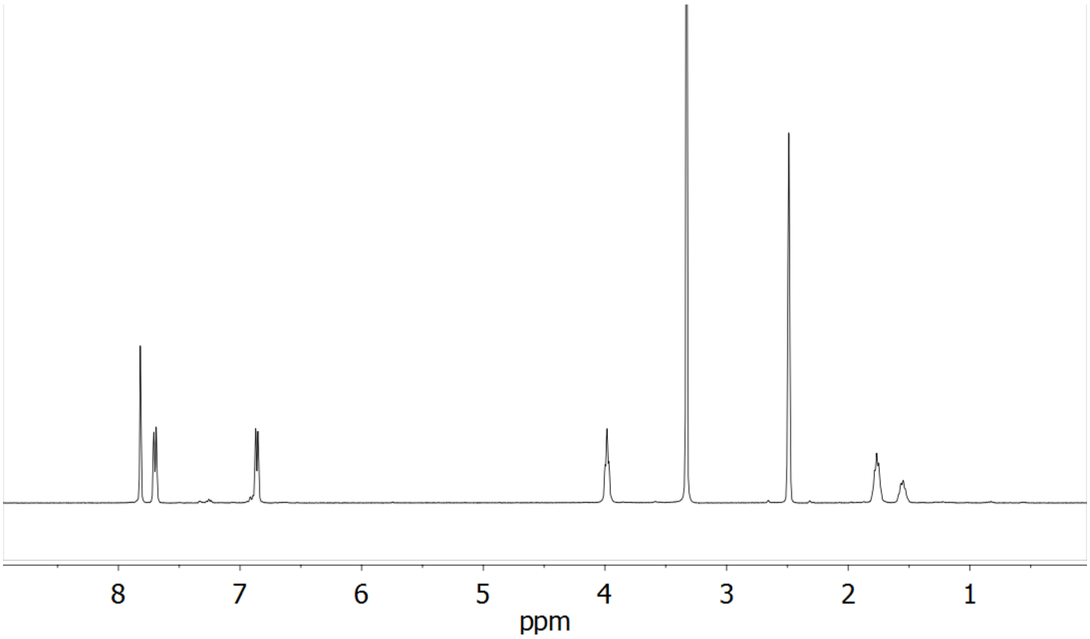

Figure S9. ^{1}H NMR spectrum of the diboronic acid **F** in DMSO-d_6.

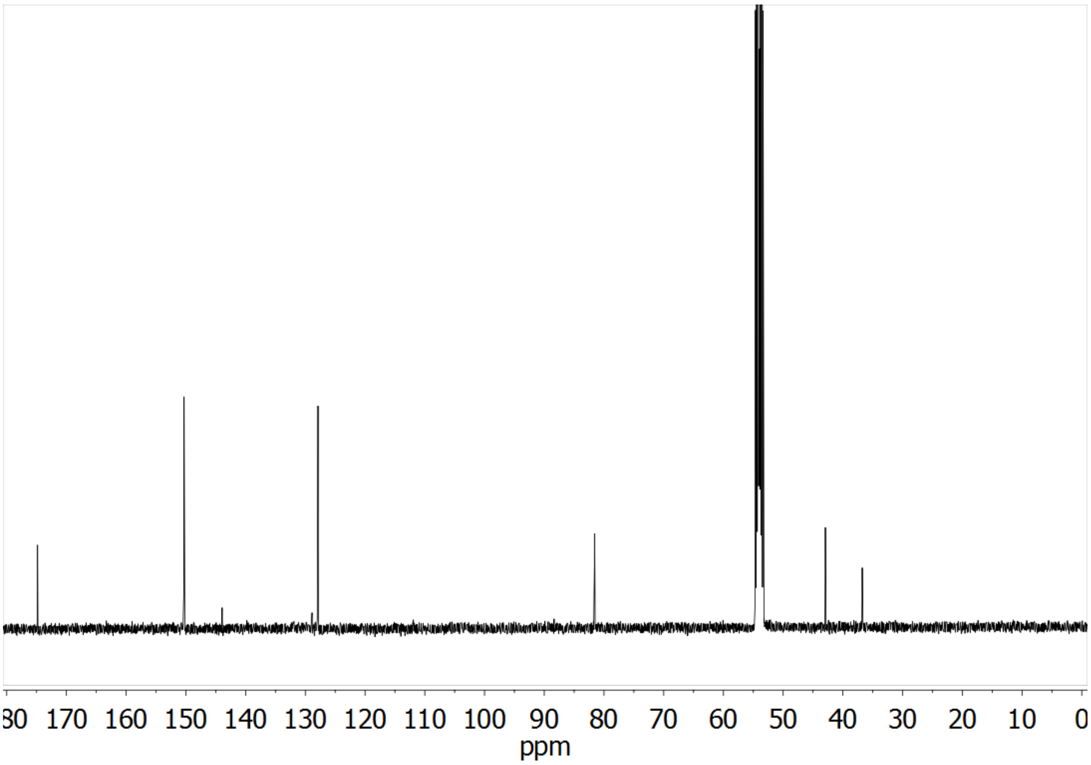

Figure S10. ^{13}C NMR spectrum of the diboronic acid **F** in DMSO-d_6.

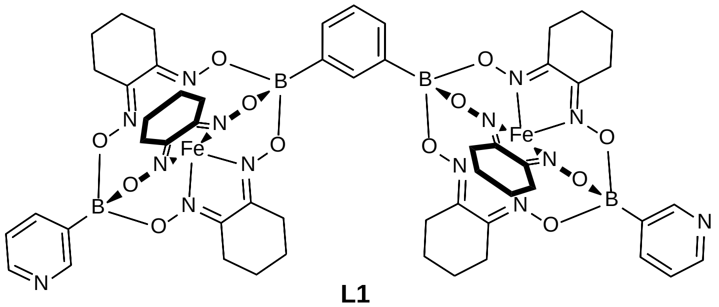

L1

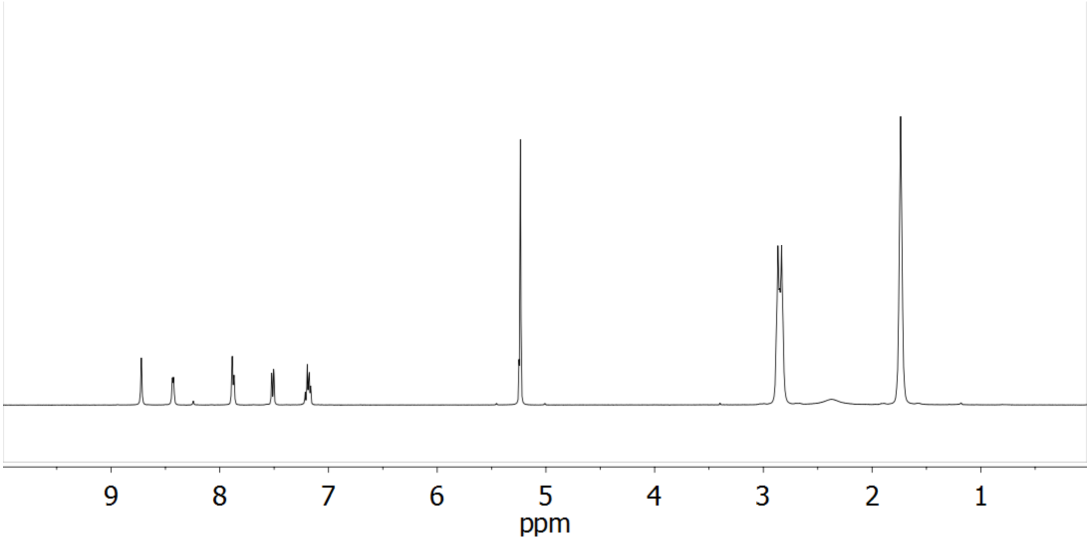

Figure S11. ^{1}H NMR spectrum of metalloligand **L1** in CD_2Cl_2.

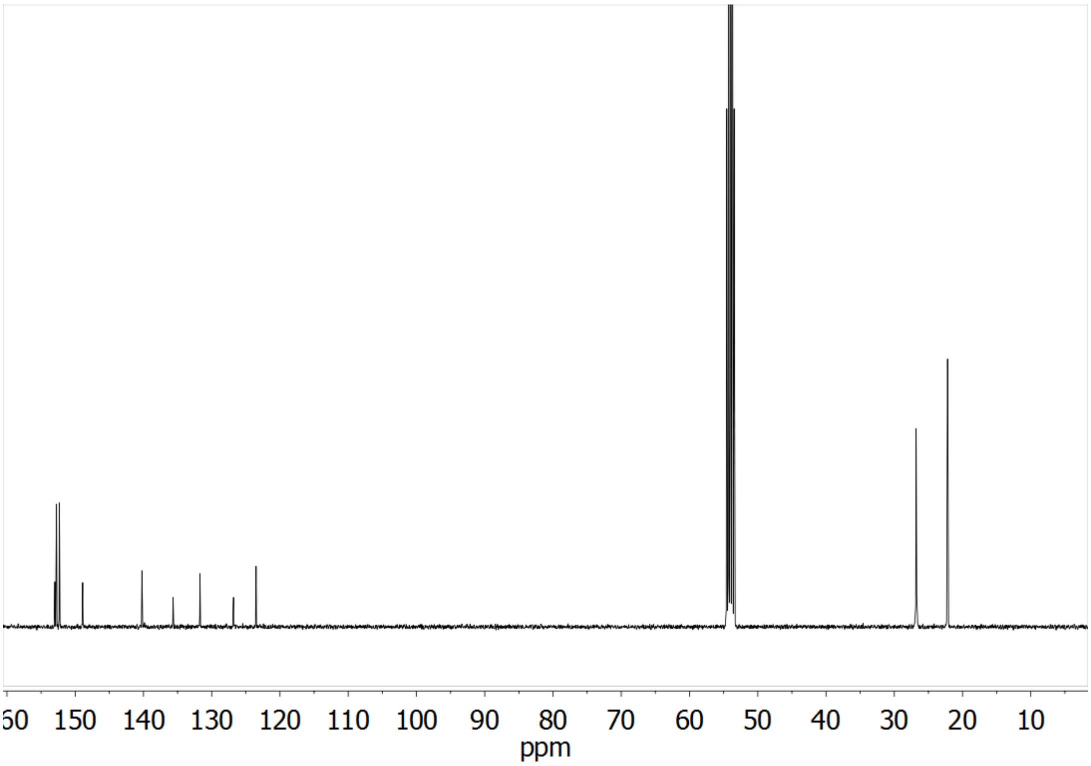

Figure S12. ^{13}C NMR spectrum of metalloligand **L1** in CD_2Cl_2.

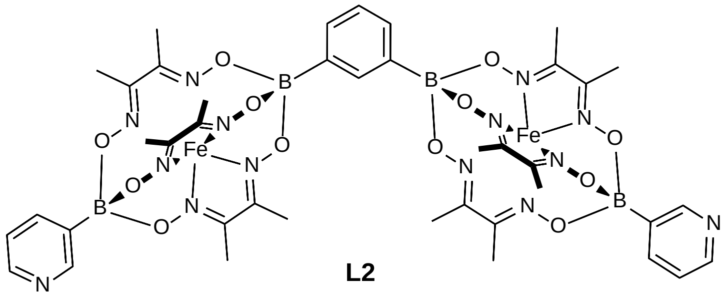

L2

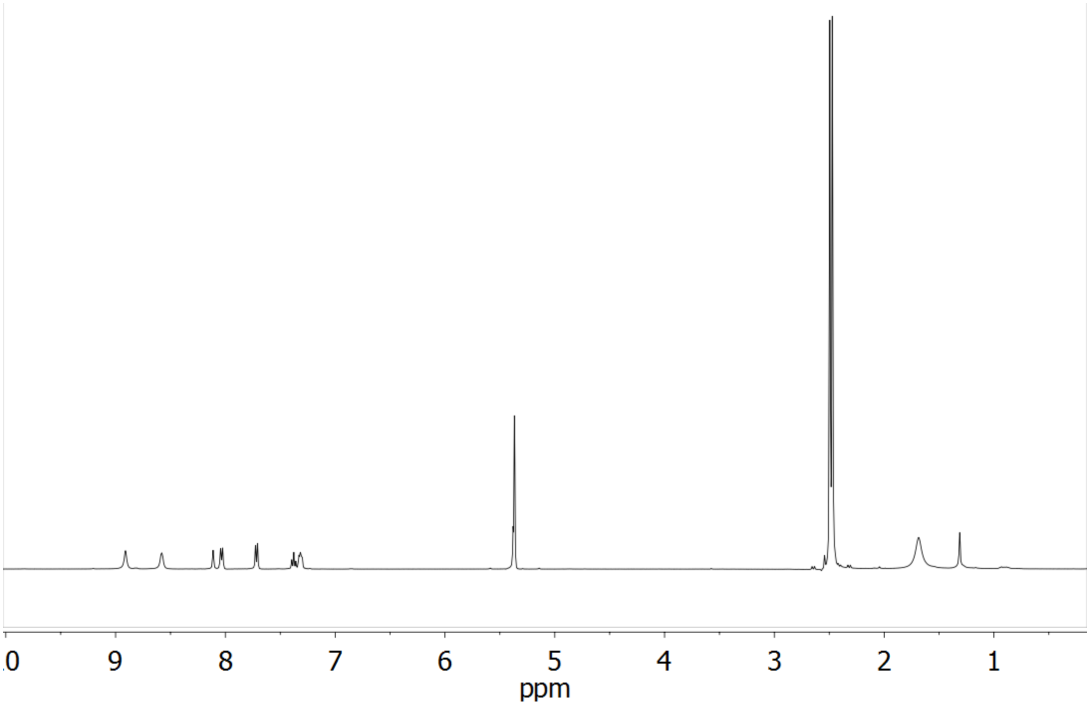

Figure S13. ^{1}H NMR spectrum of metalloligand **L2** in CD_2Cl_2.

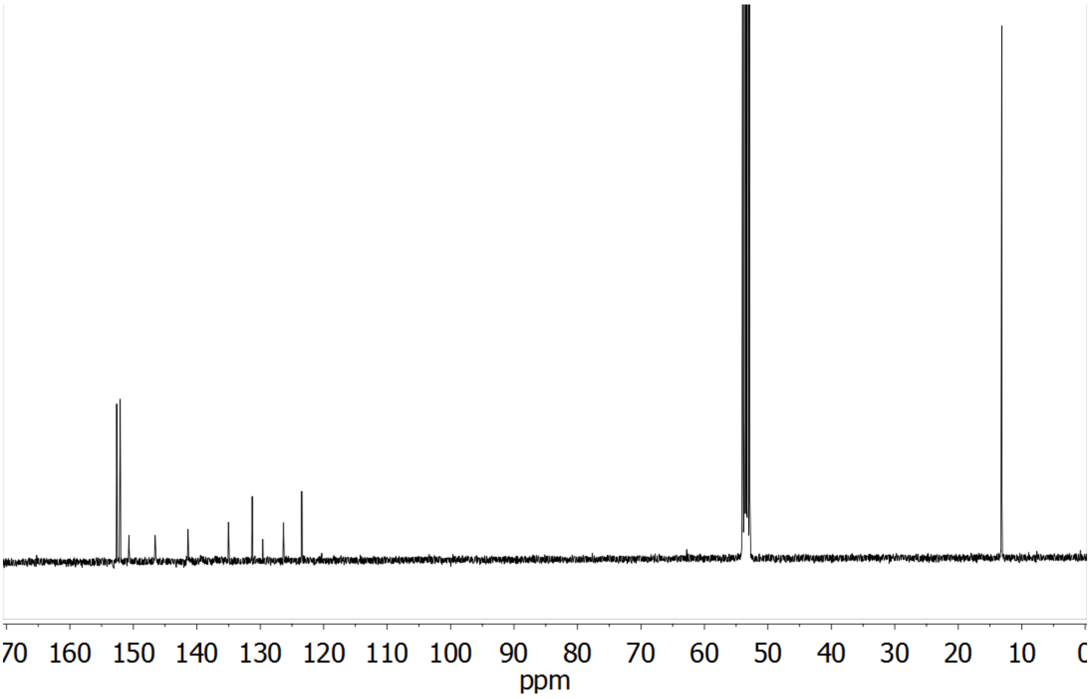

Figure S14. ^{13}C NMR spectrum of metalloligand **L2** in CD_2Cl_2.

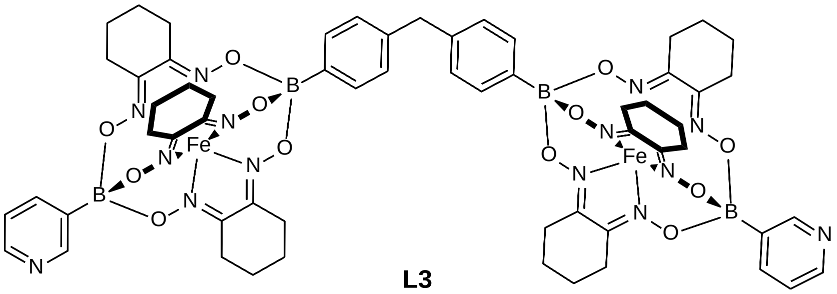

L3

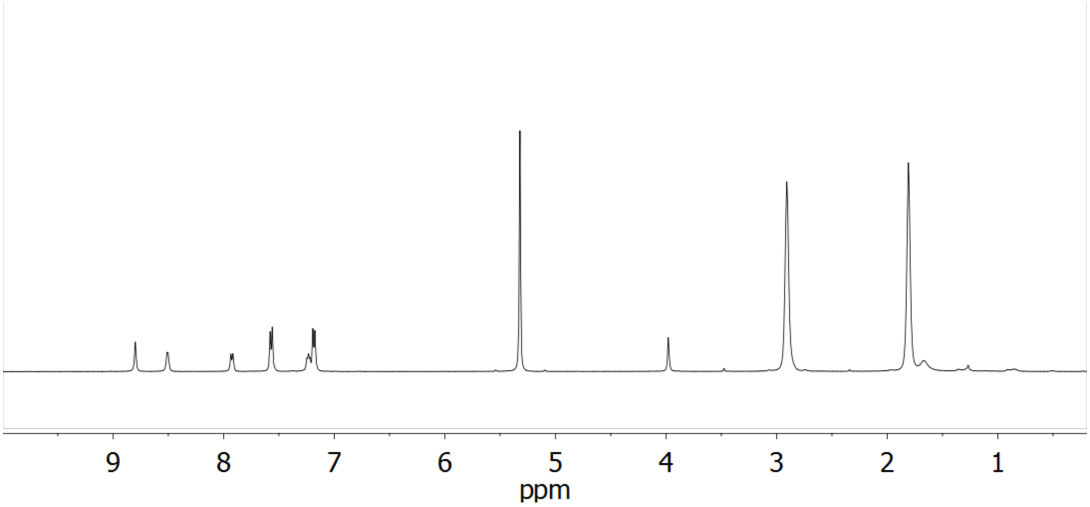

Figure S15. ^{1}H NMR spectrum of metalloligand **L3** in CD_2Cl_2.

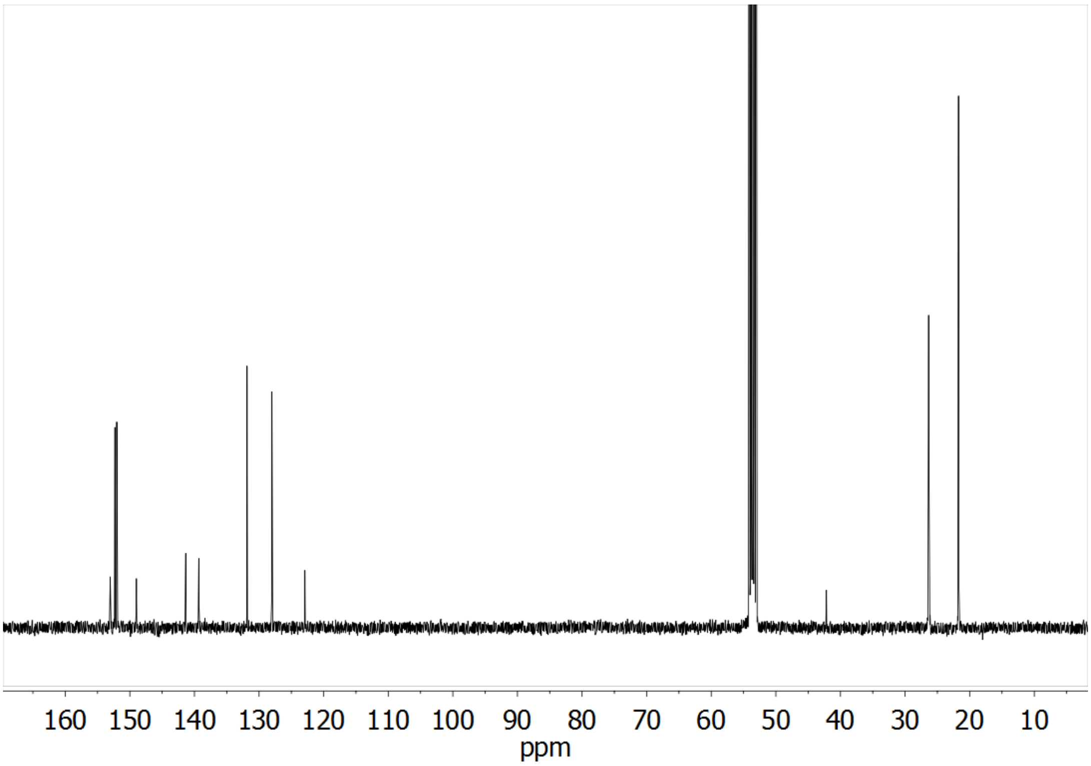

Figure S16. ^{13}C NMR spectrum of metalloligand **L3** in CD_2Cl_2.

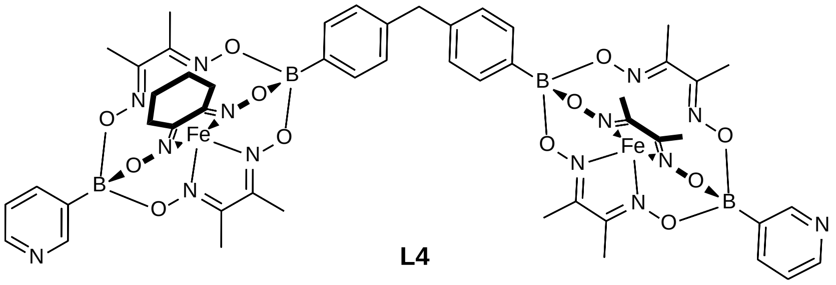

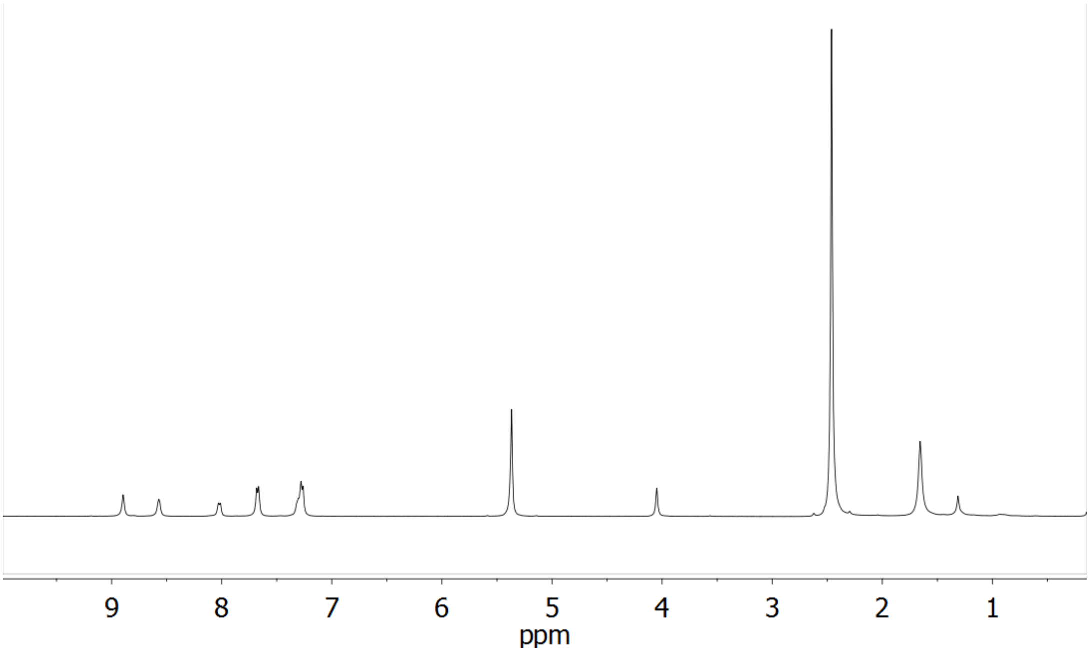

Figure S17. ^{1}H NMR spectrum of metalloligand **L4** in CD_2Cl_2.

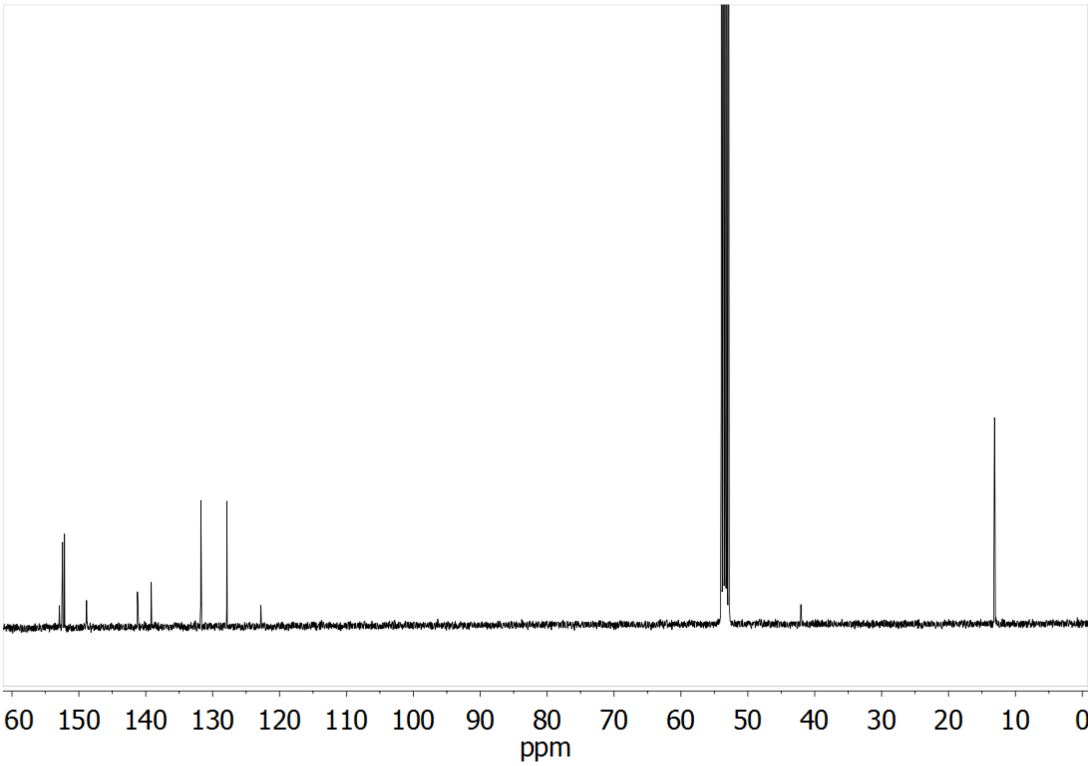

Figure S18. ^{13}C NMR spectrum of metalloligand **L4** in CD_2Cl_2.

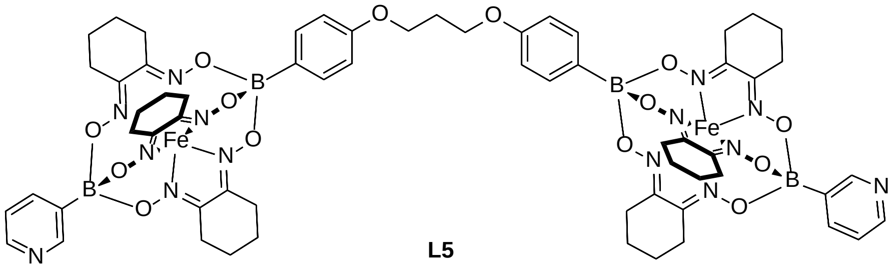

L5

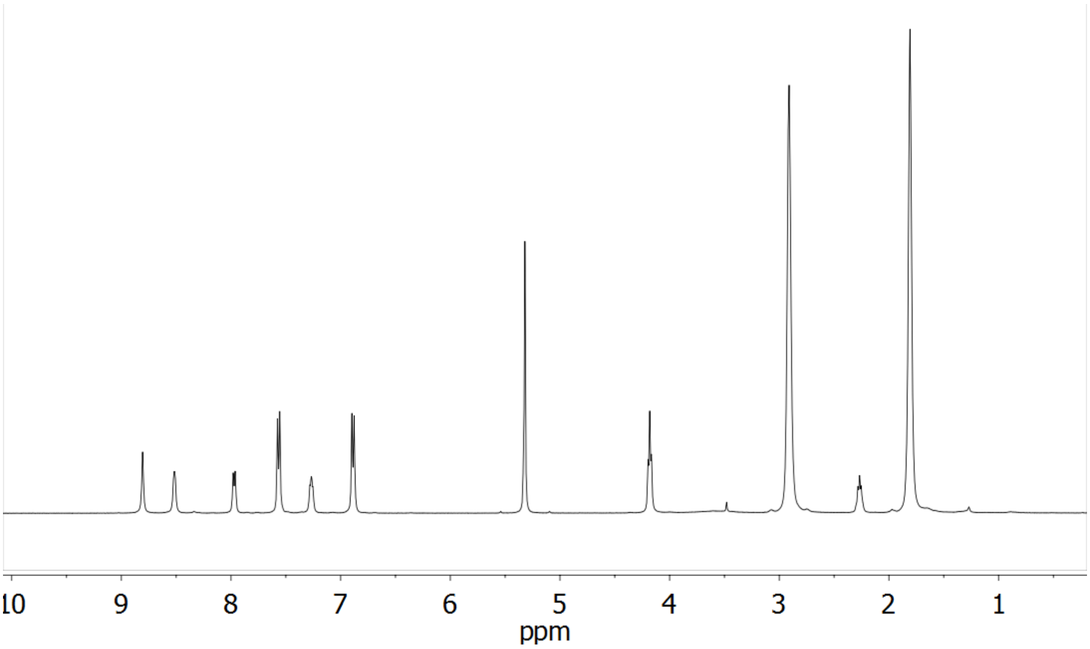

Figure S19. ^{1}H NMR spectrum of metalloligand **L5** in CD_2Cl_2.

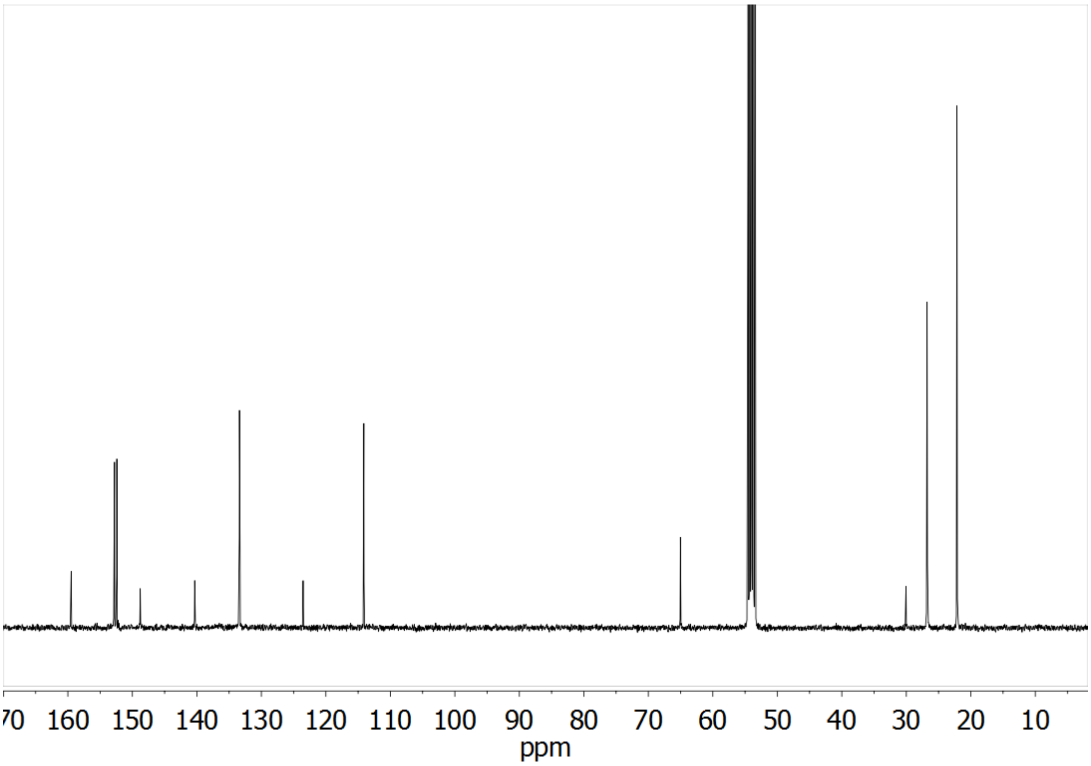

Figure S20. ^{13}C NMR spectrum of metalloligand **L5** in CD_2Cl_2.

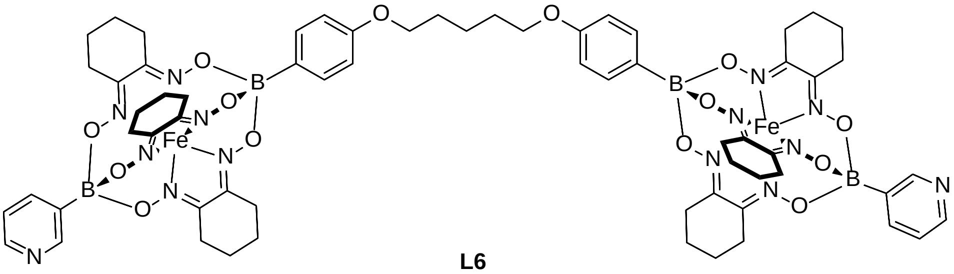

L6

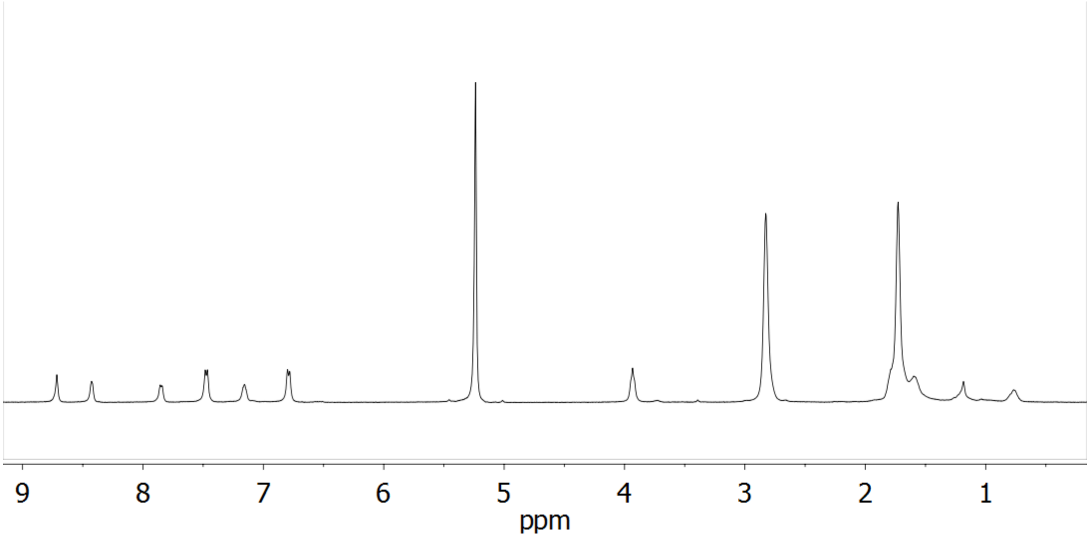

Figure S21. ^{1}H NMR spectrum of metalloligand **L6** in CD_2Cl_2.

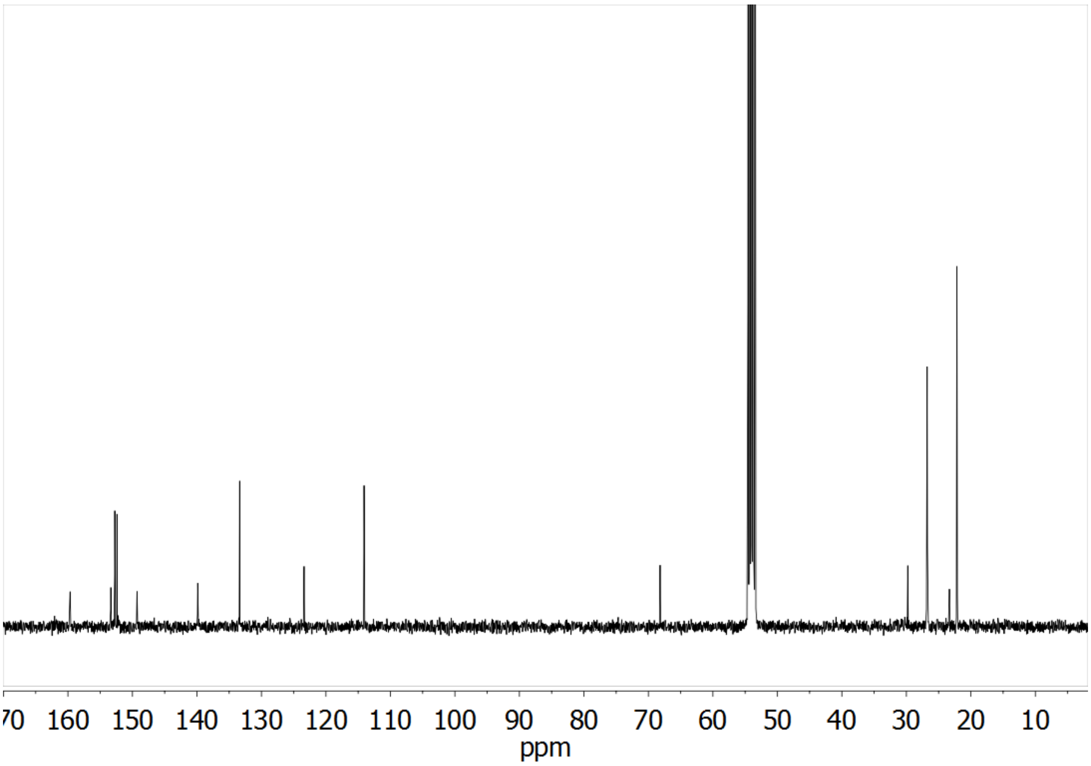

Figure S22. ^{13}C NMR spectrum of metalloligand **L6** in CD_2Cl_2.

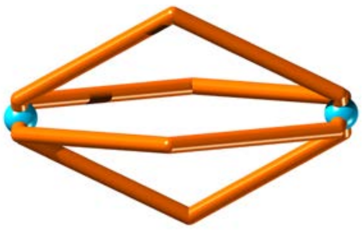

Cage 1: $[Pd^{II}_2(\mathbf{L1})_4](BF_4)_4$

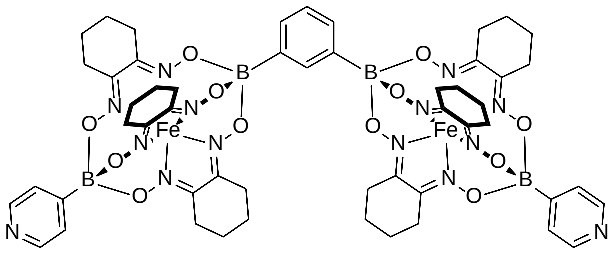

from ligand **L1**

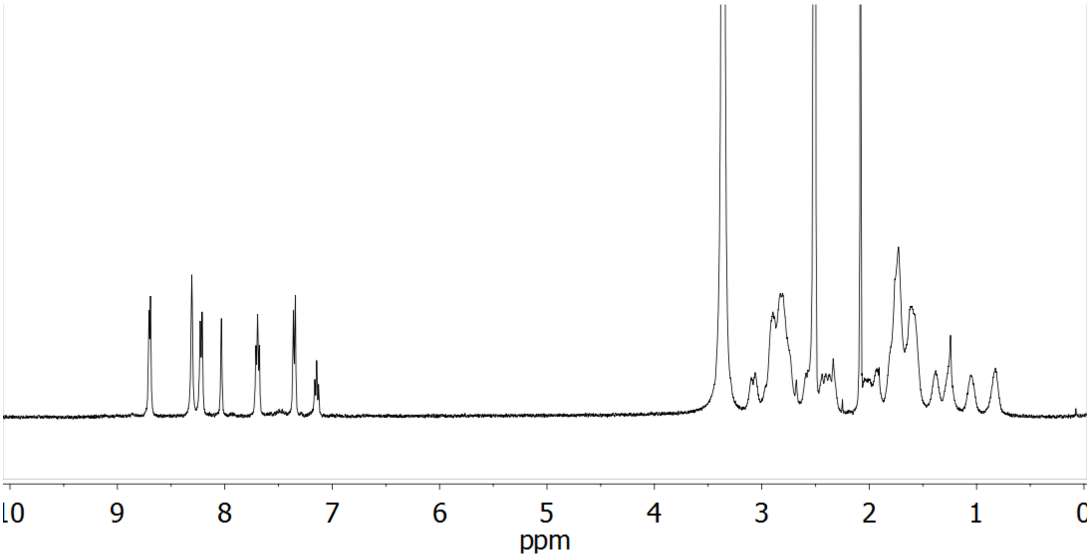

Figure S23. ¹H NMR spectrum of coordination cage **1** in DMSO-d_6.

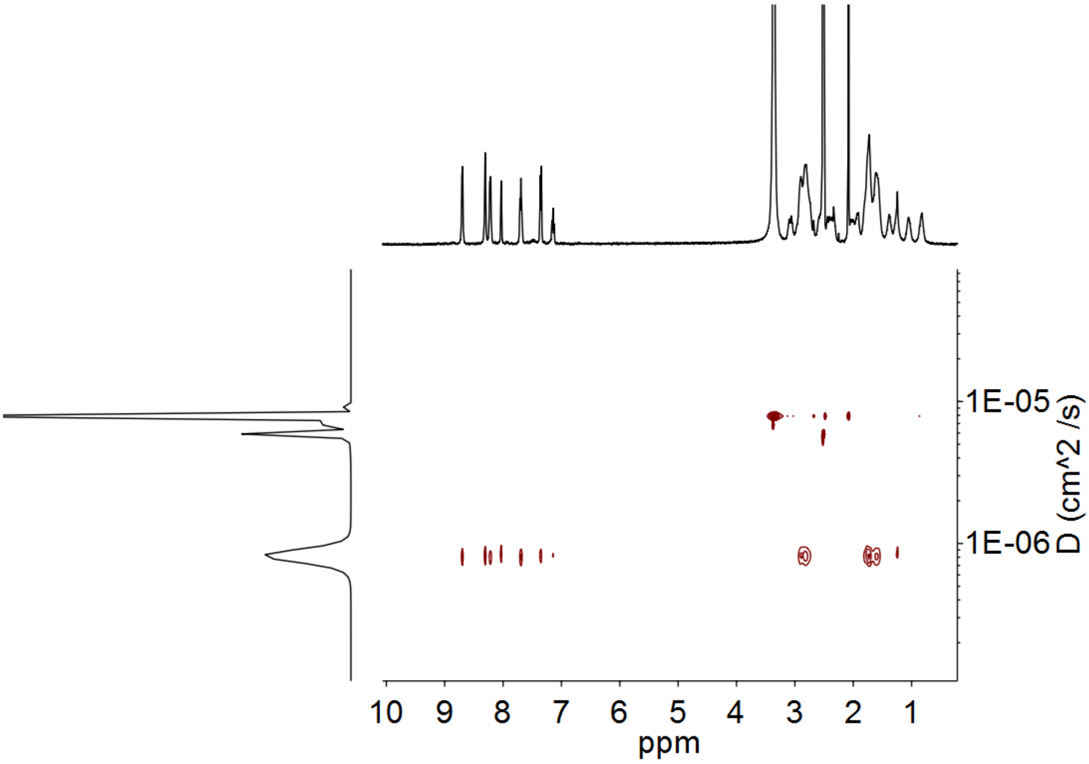

Figure S24. ¹H DOSY NMR spectrum of coordination cage **1** in DMSO-d_6.

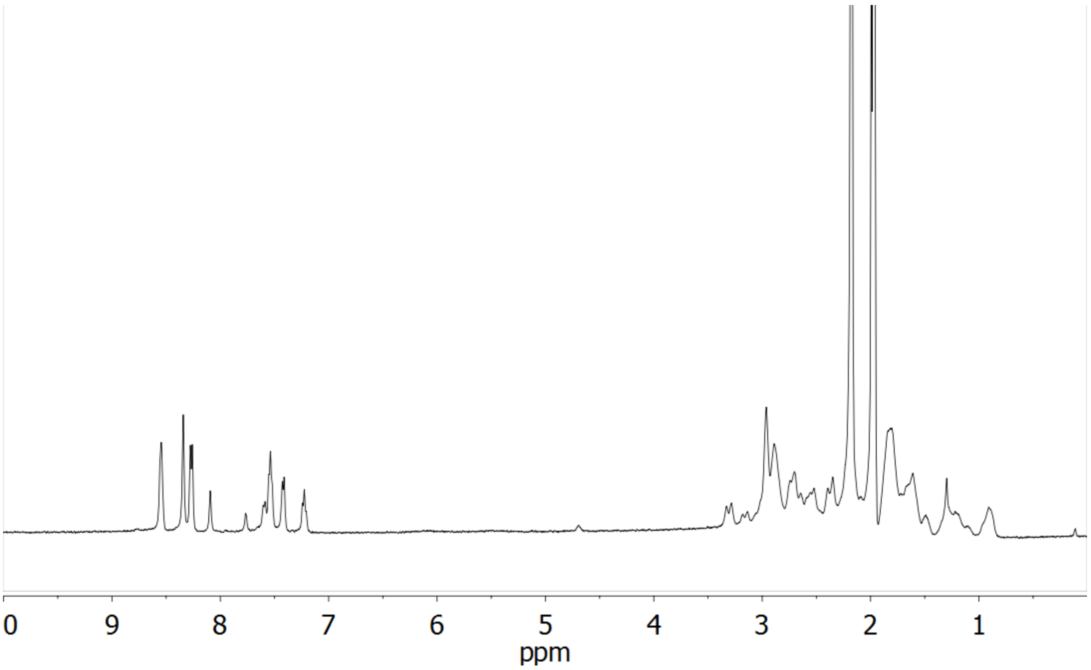

Figure S25. ^{1}H NMR spectrum of coordination cage **1** in CD_3CN.

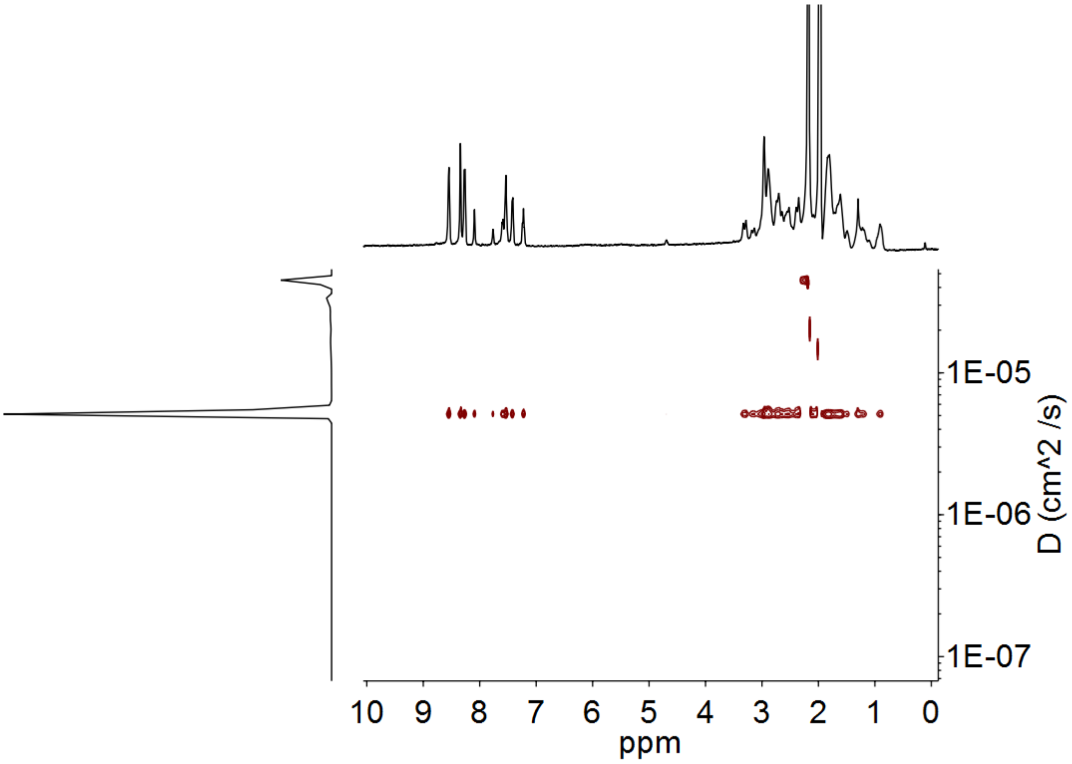

Figure S26. ^{1}H DOSY NMR spectrum of coordination cage **1** in CD_3CN.

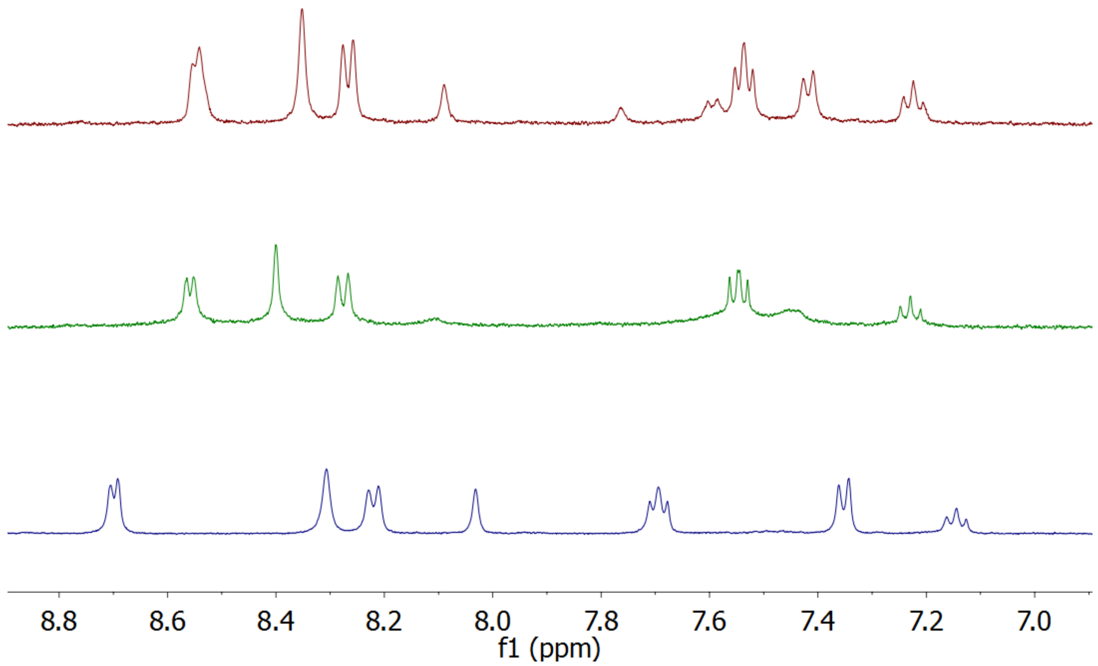

Figure S27. ^{1}H NMR stack plot of 3 spectra of coordination cage **1**. Top: **1** in CD_3CN at 298 K, middle: **1** in CD_3CN at 328 K and for reference the bottom spectrum shows **1** in DMSO-d_6 at 298 K.

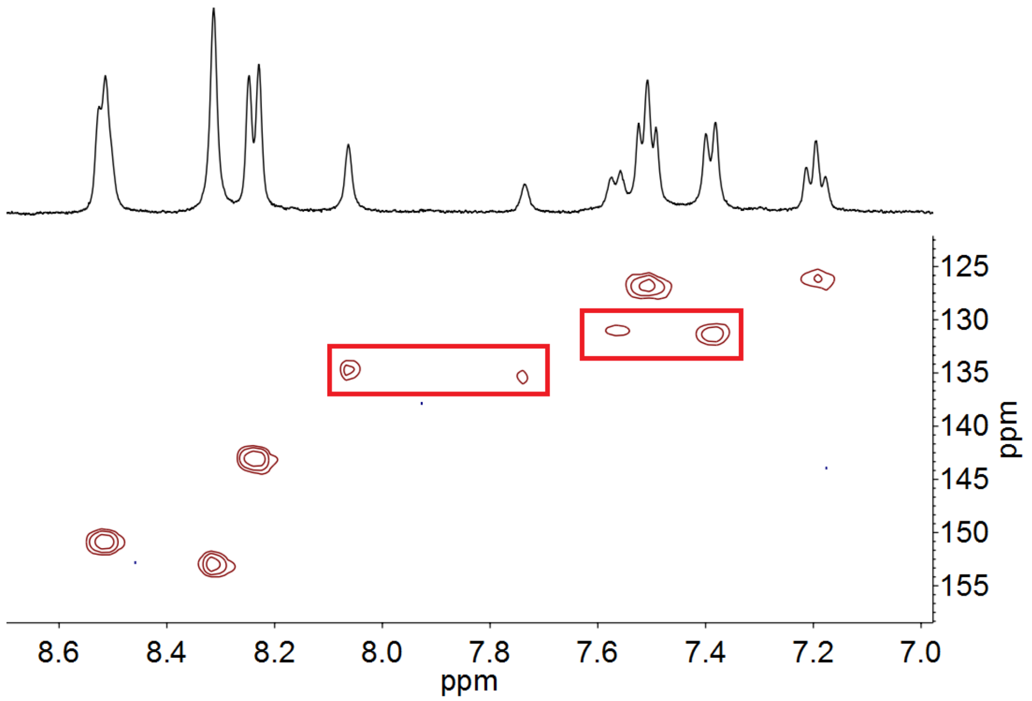

Figure S28. HSQC NMR spectrum of cage **1** in CD_3CN at 298 K.

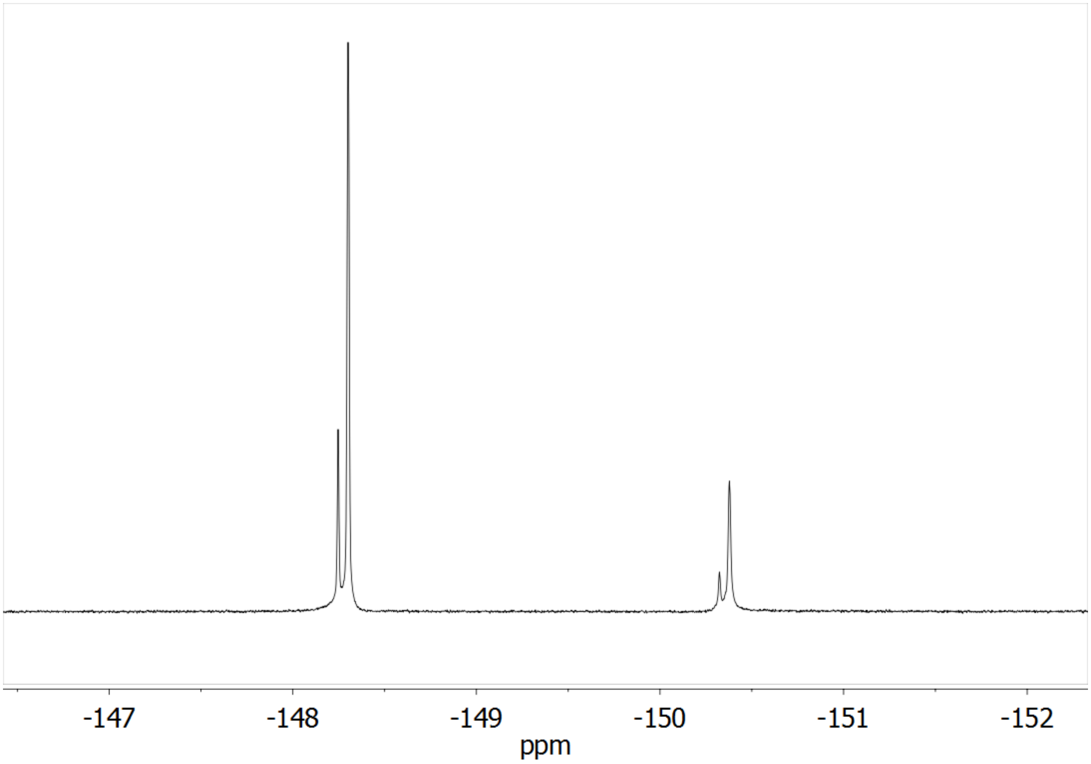

Figure S29. ^{19}F NMR spectrum of cage **1** in DMSO-d_6.

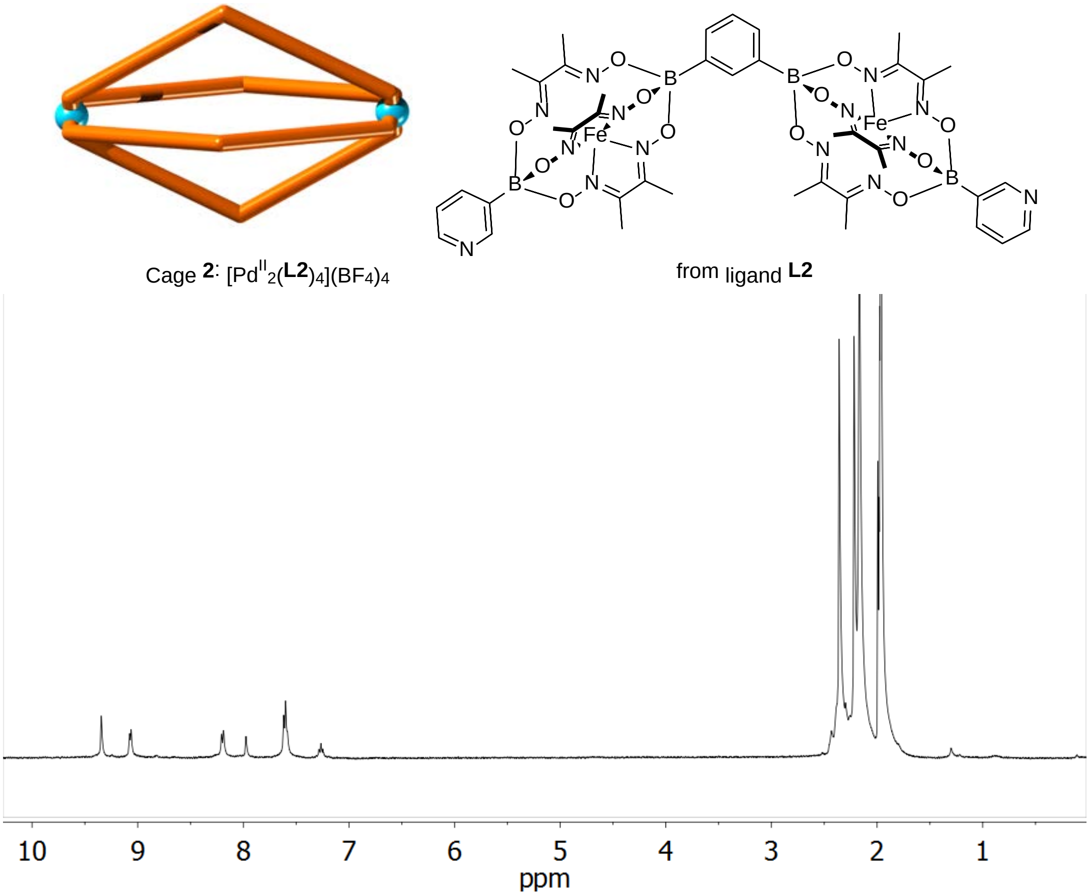

Figure S30. ^{1}H NMR spectrum of coordination cage **2** in CD_3CN.

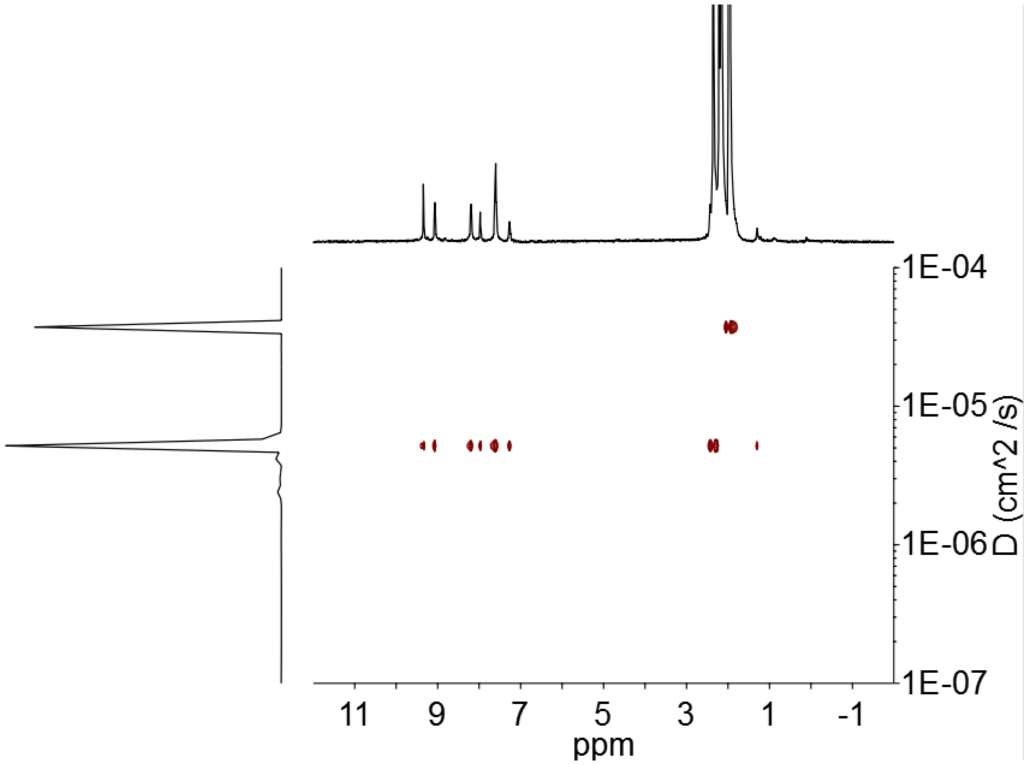

Figure S31. ^{1}H DOSY NMR spectrum of coordination cage **2** in CD_3CN.

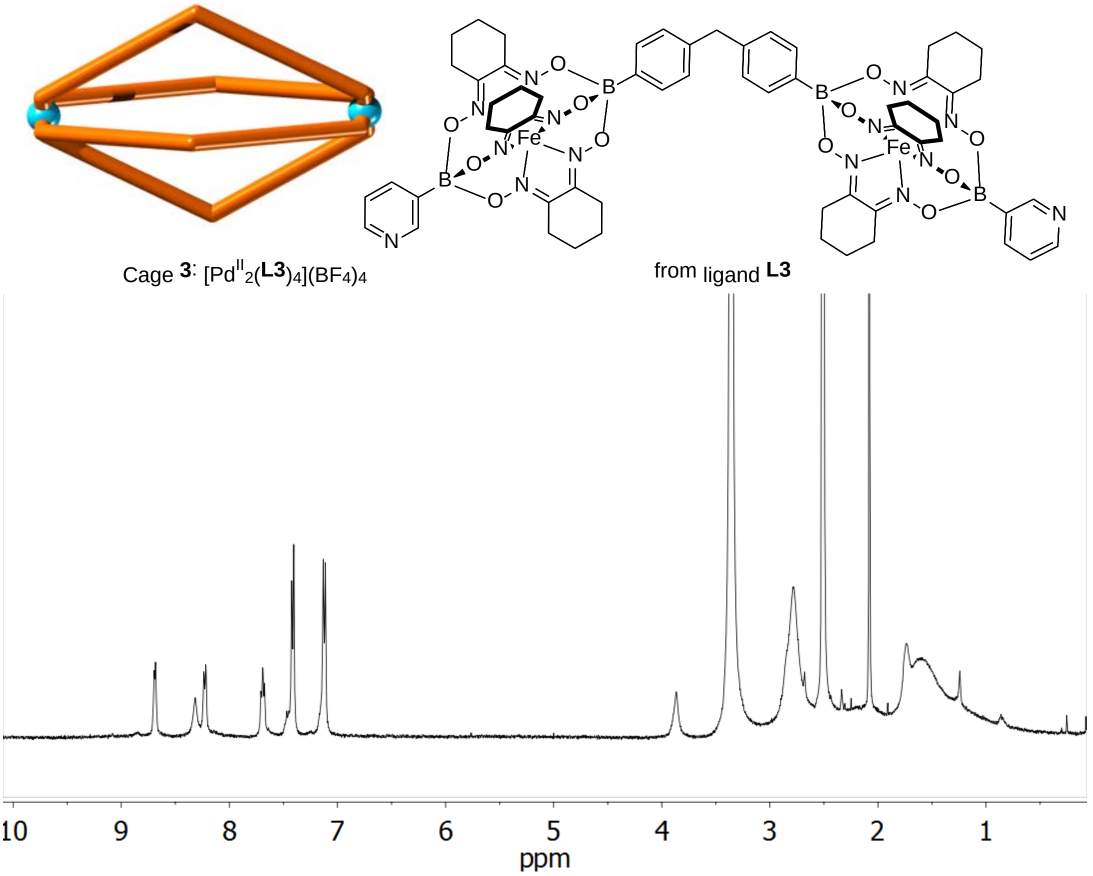

Figure S32. ^{1}H NMR spectrum of coordination cage **3** in DMSO-d_6.

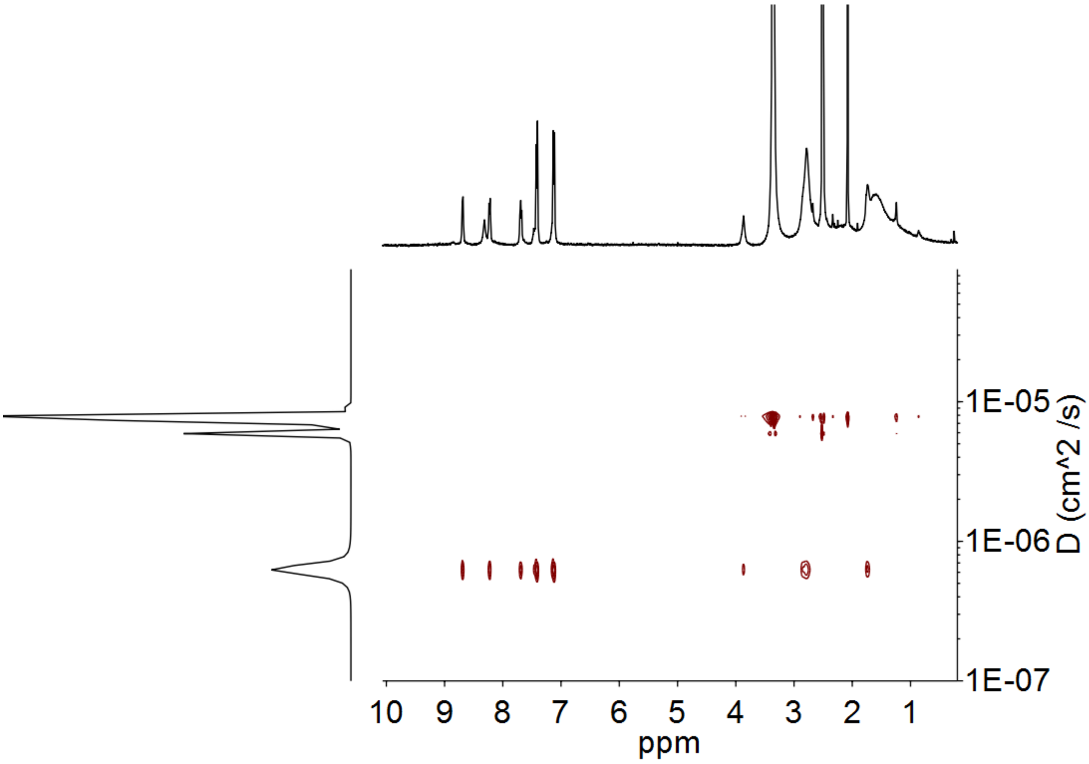

Figure S33. ^{1}H DOSY NMR spectrum of coordination cage **3** in DMSO-d_6.

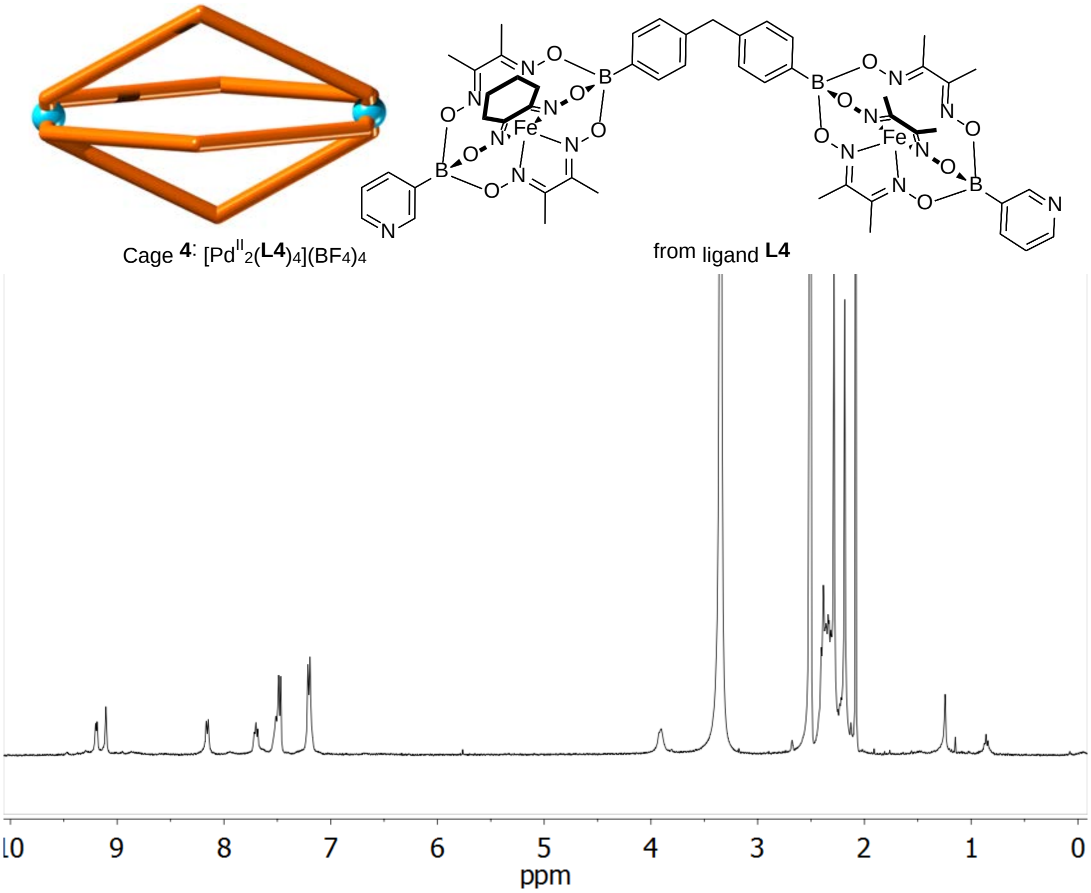

Figure S34. ^{1}H NMR spectrum of coordination cage **4** in DMSO-d_6.

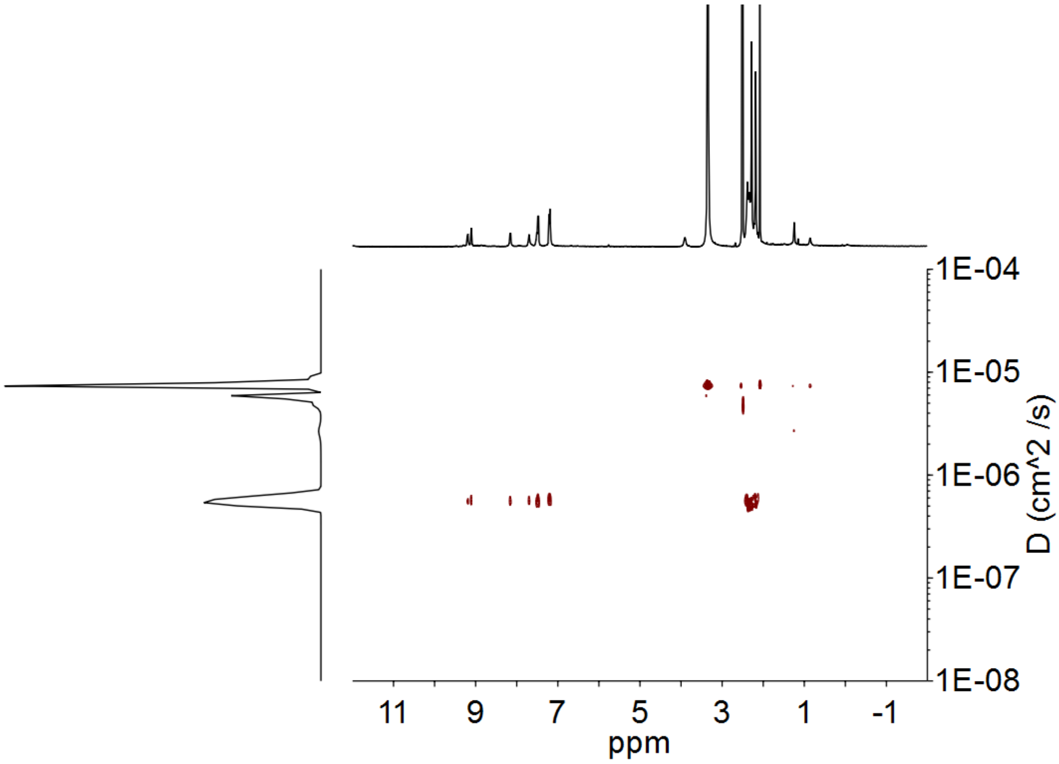

Figure S35. ^{1}H DOSY NMR spectrum of coordination cage **4** in DMSO-d_6.

4. Mass spectra Pd_2L_4 coordination cages

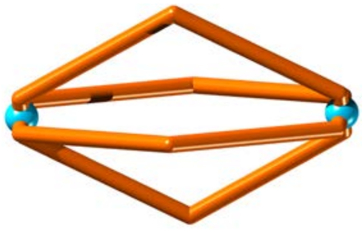

Cage **1**: $[Pd^{II}_2(\mathbf{L1})_4](BF_4)_4$

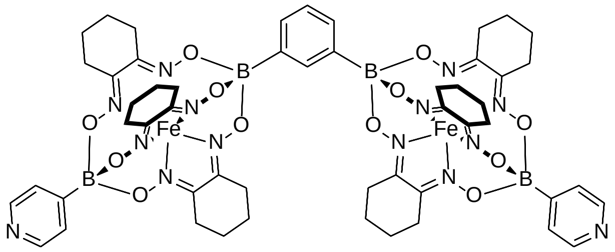

from ligand **L1**

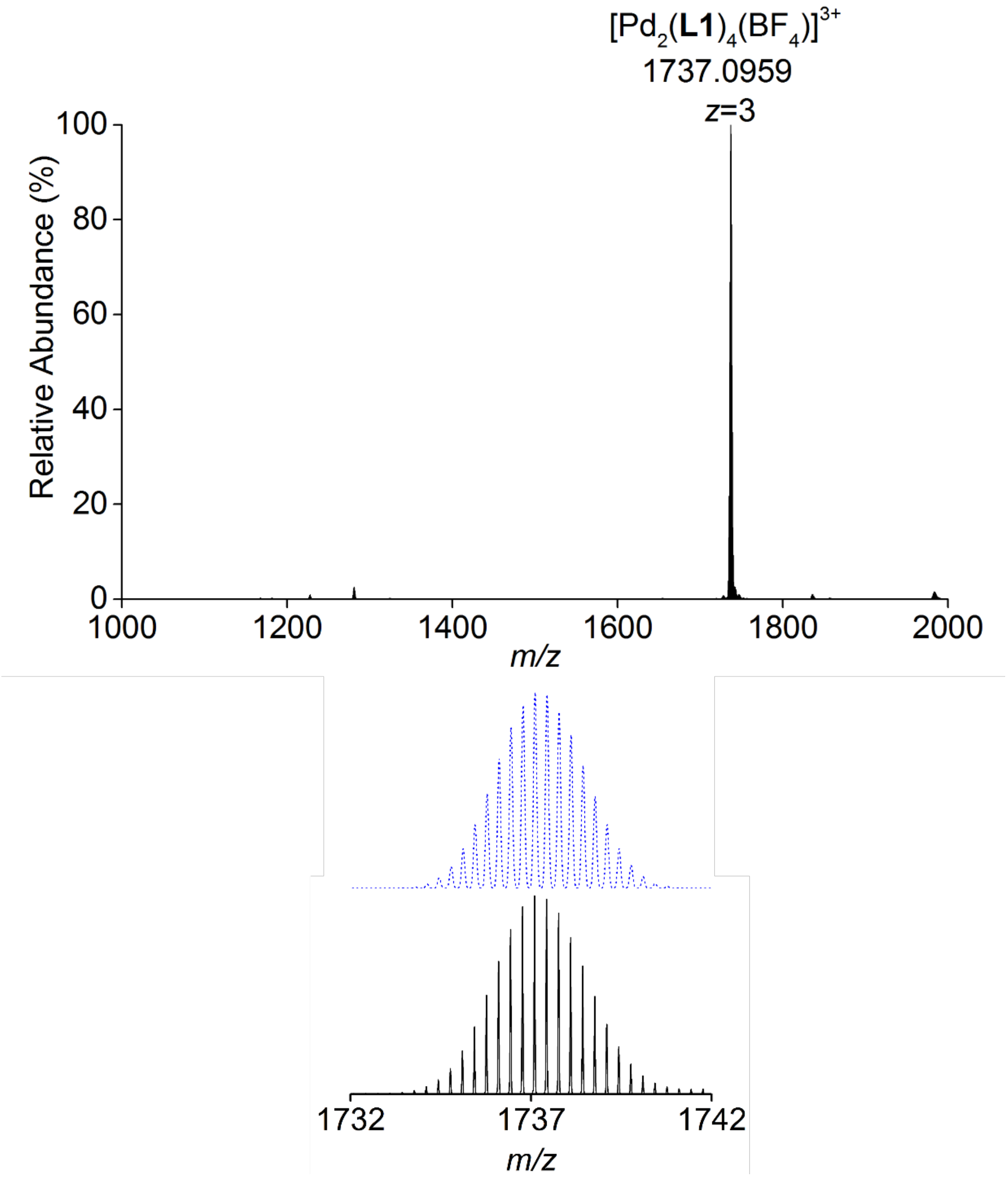

Figure S36. HRMS spectrum of coordination cage **1** in CH_3CN (top). Zoom-in of the peak at 1737 *m/z* with simulated spectrum shown in blue (bottom).

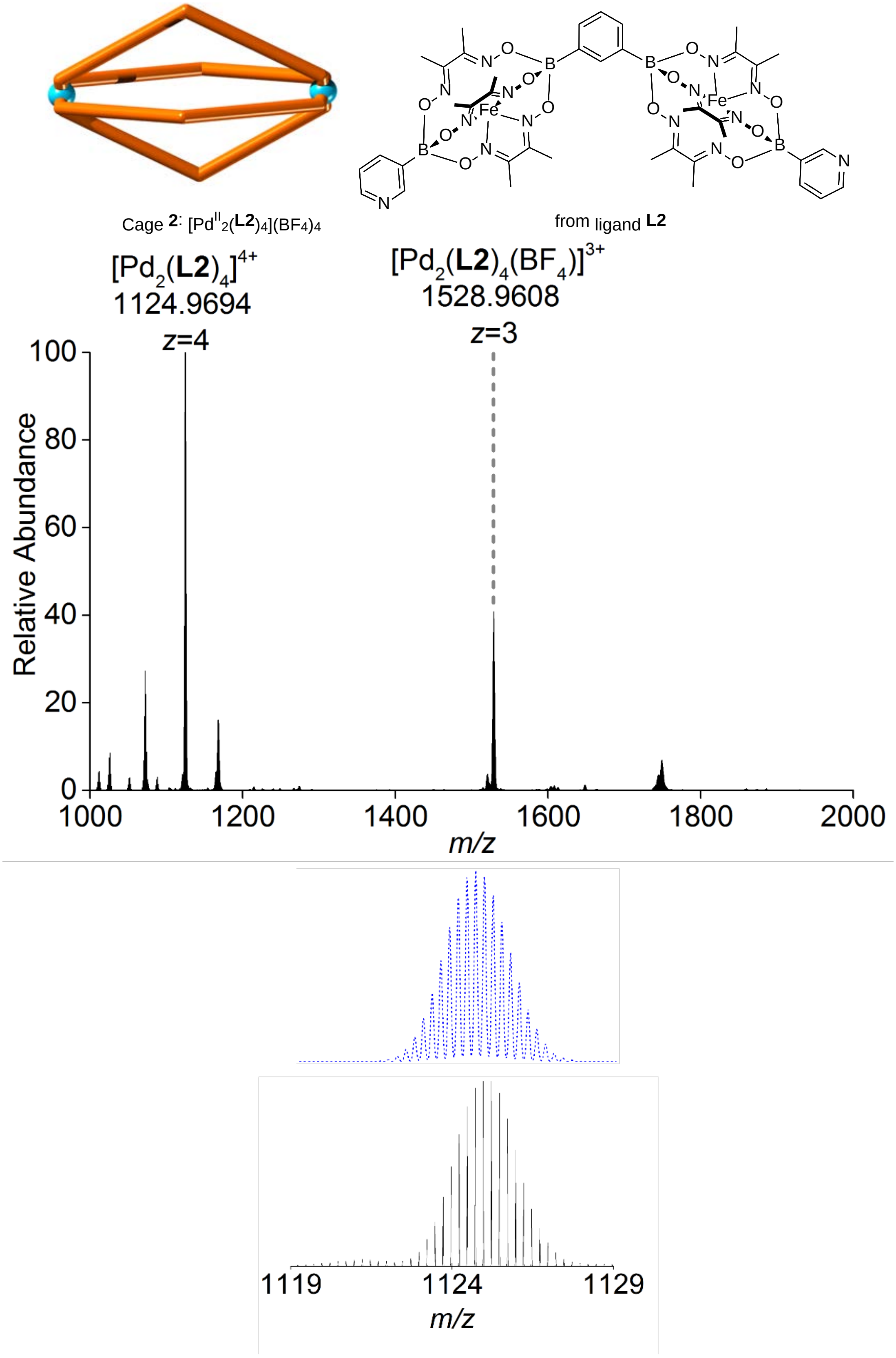

Figure S37. HRMS spectrum of coordination cage **2** in CH_3CN (top). Zoom-in of the peak at 1125 *m/z* with simulated spectrum shown in blue (bottom).

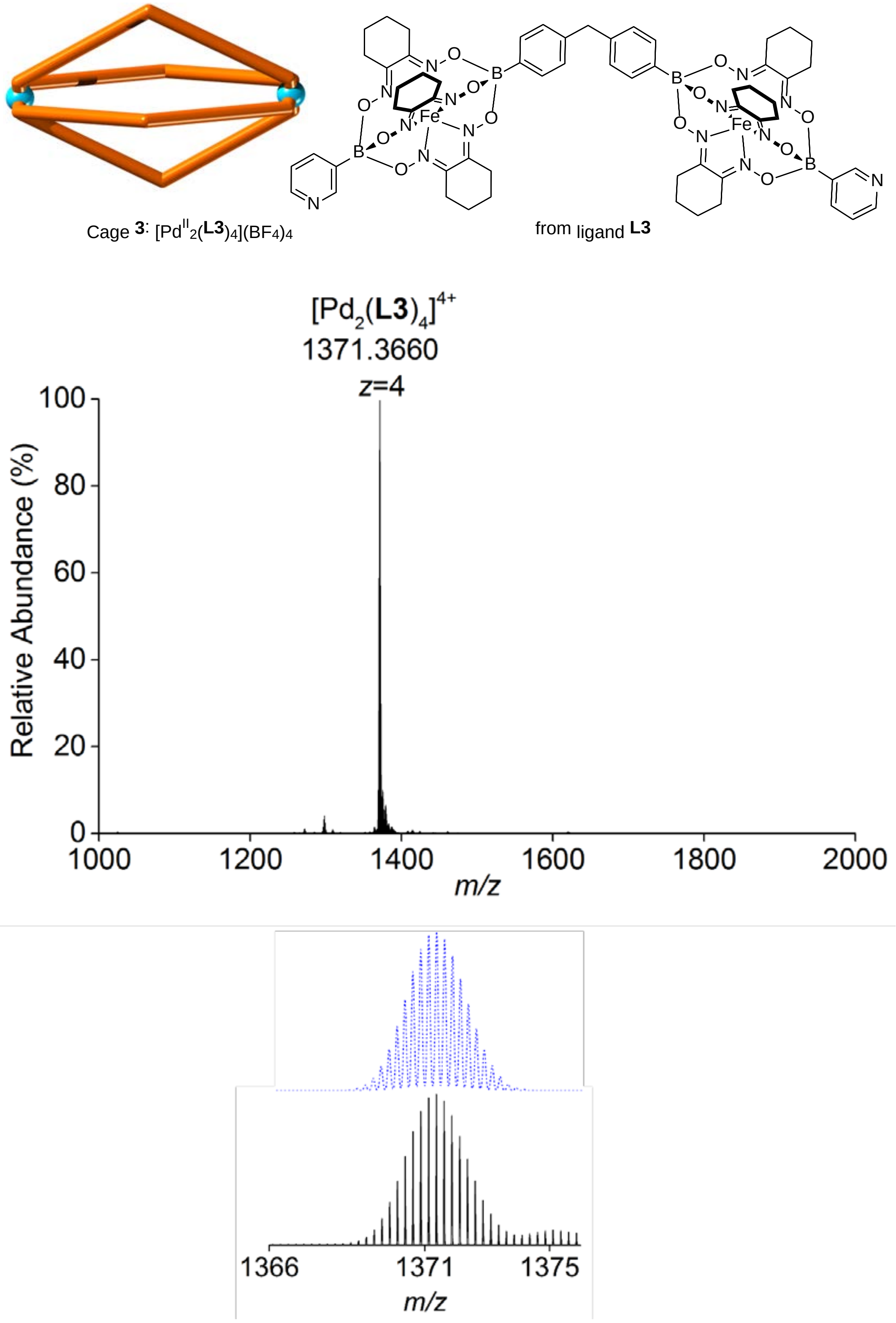

Figure S38. HRMS spectrum of coordination cage **3** in DMSO, a few drops of which were added to CH_3CN (top) to record the spectrum. Zoom-in of the peak at 1371 *m/z* with simulated spectrum shown in blue (bottom).

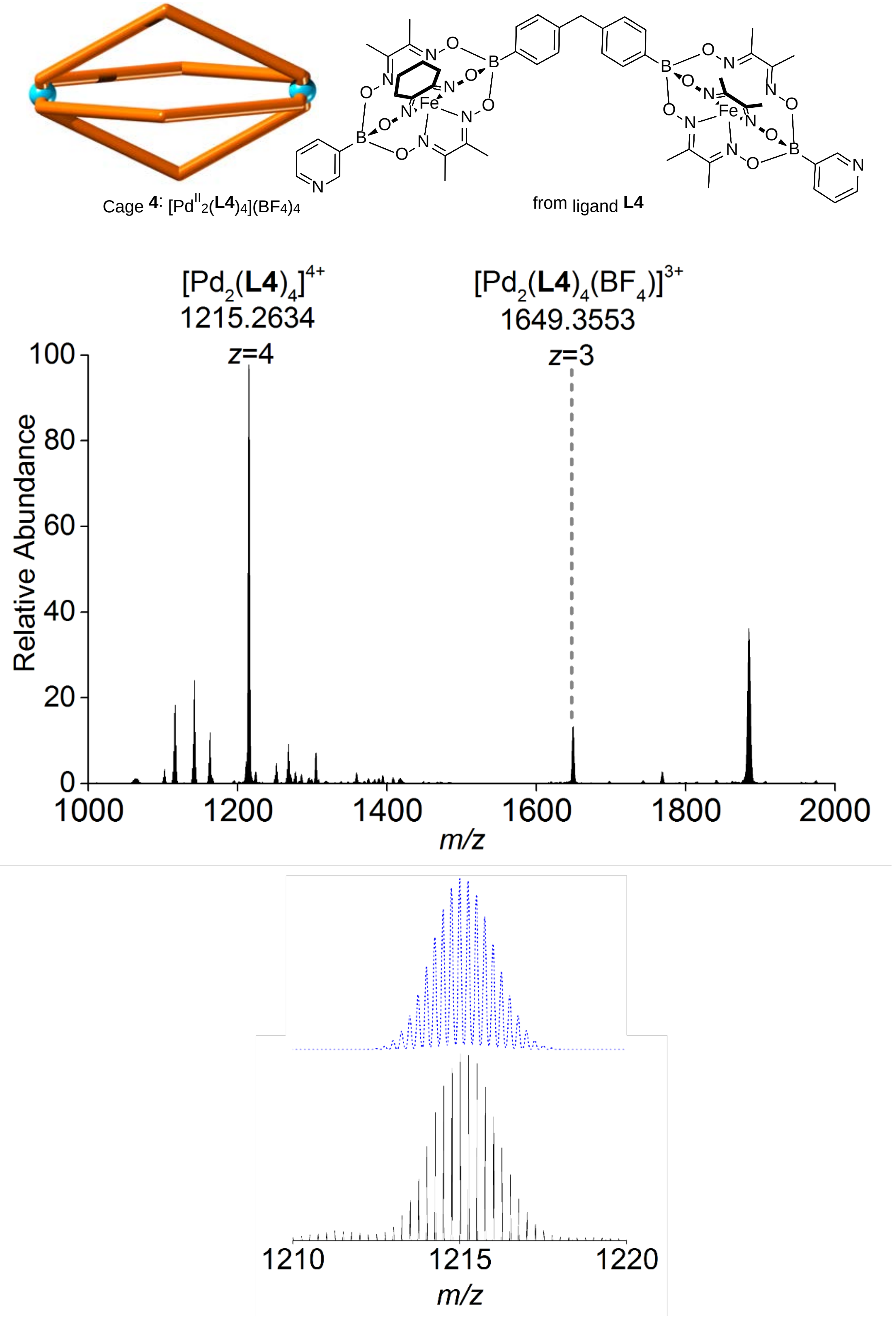

Figure S39. HRMS spectrum of coordination cage **4** in DMSO, a few drops of which were added to CH_3CN (top) to record the spectrum. Zoom-in of the peak at 1215 *m/z* with simulated spectrum shown in blue (bottom).

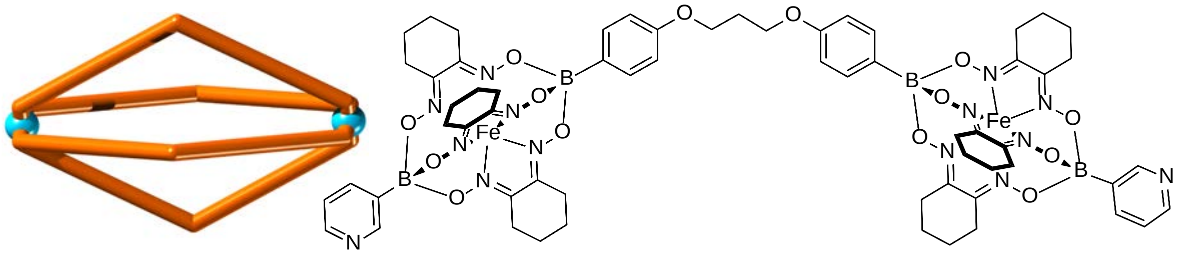

Cage **5**: $[Pd^{II}_2(\mathbf{L5})_4](BF_4)_4$ from ligand **L5**

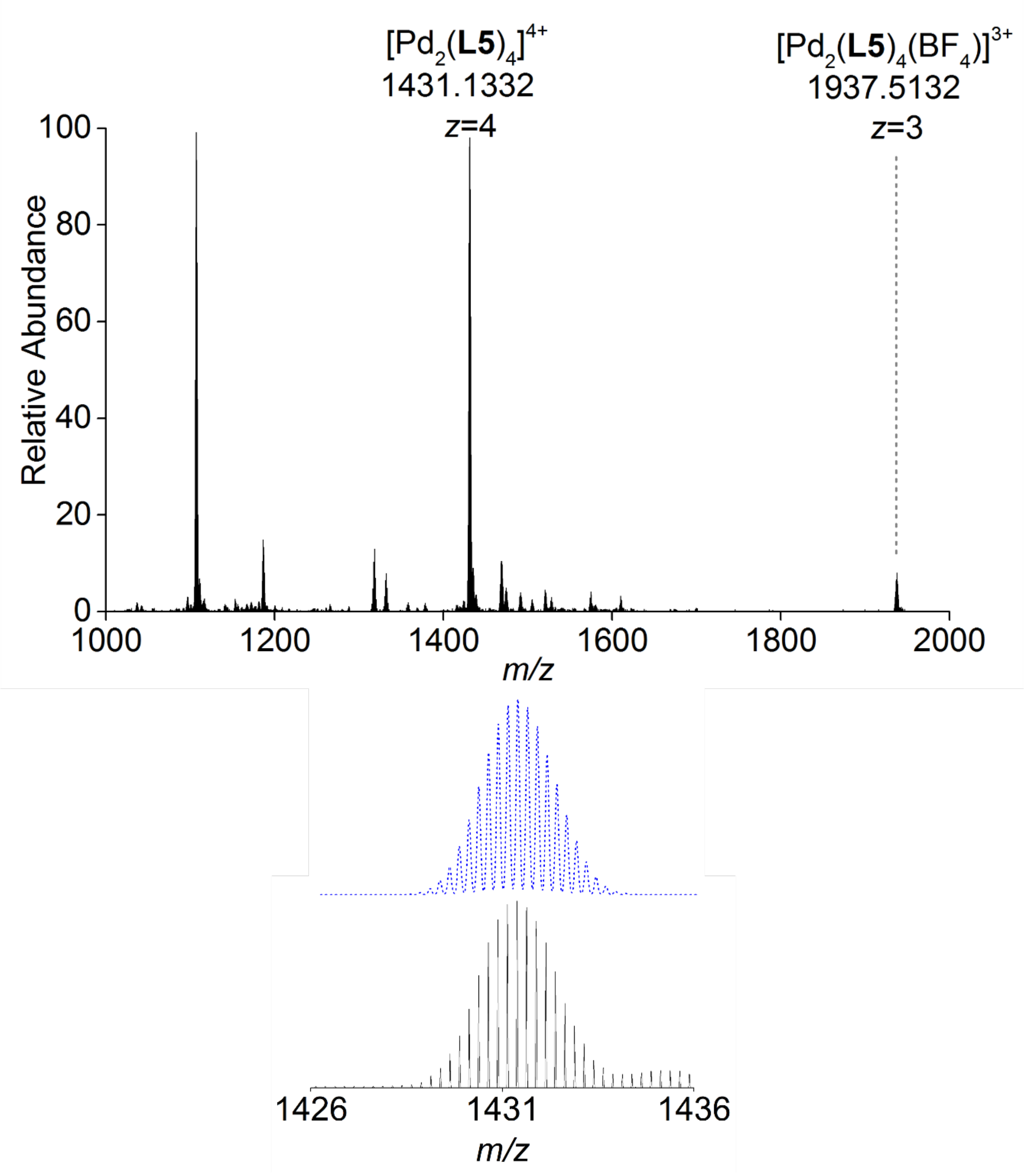

Figure S40. HRMS spectrum of coordination cage **5** in DMSO, a few drops of which were added to CH_3CN (top) to record the spectrum. Zoom-in of the peak at 1431 *m/z* with simulated spectrum shown in blue (bottom).

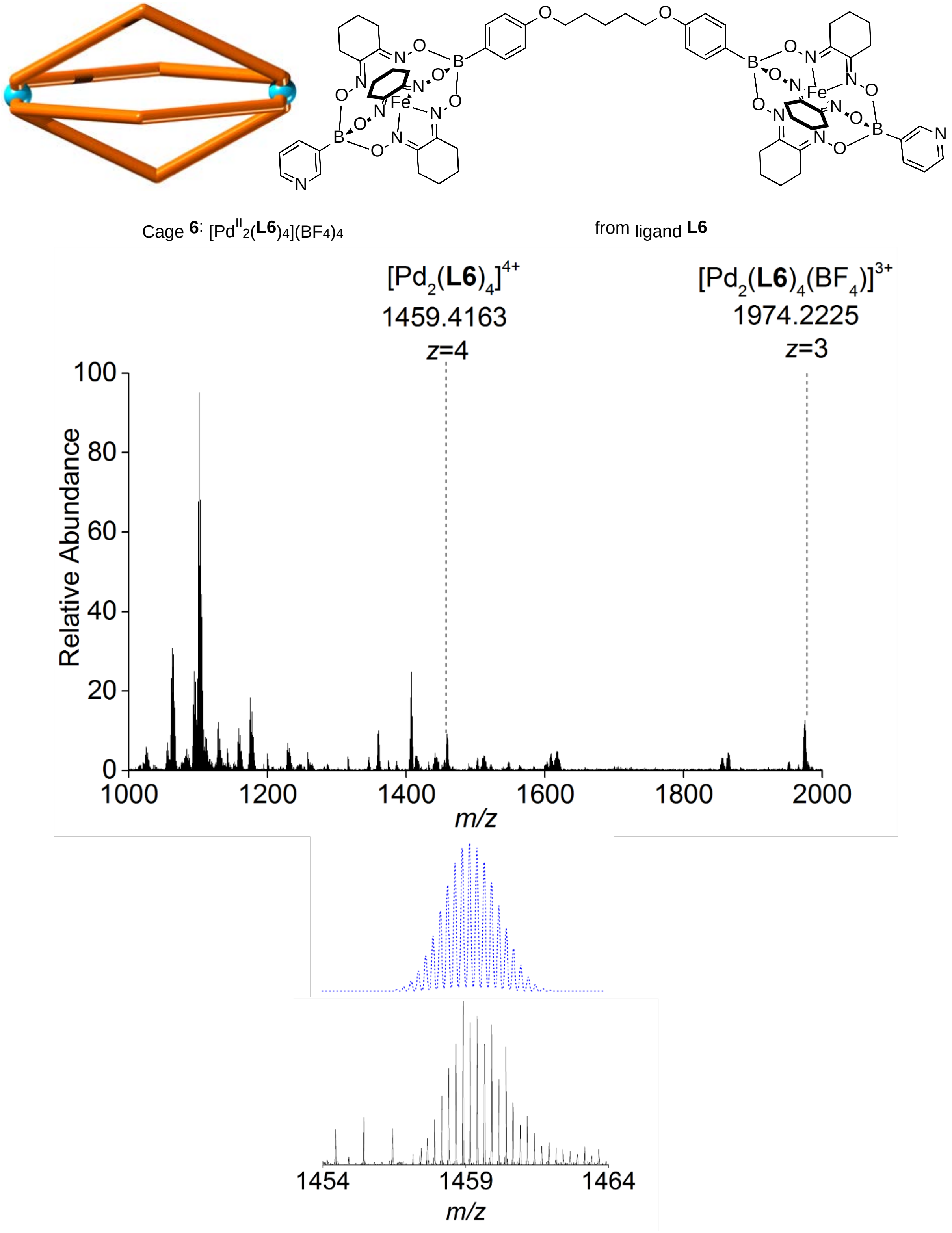

Figure S41. HRMS spectrum of coordination cage **6** in DMSO, a few drops of which were added to CH_3CN (top) to record the spectrum. Zoom-in of the peak at 1459 *m/z* with simulated spectrum shown in blue (bottom).

5. Destruction experiments

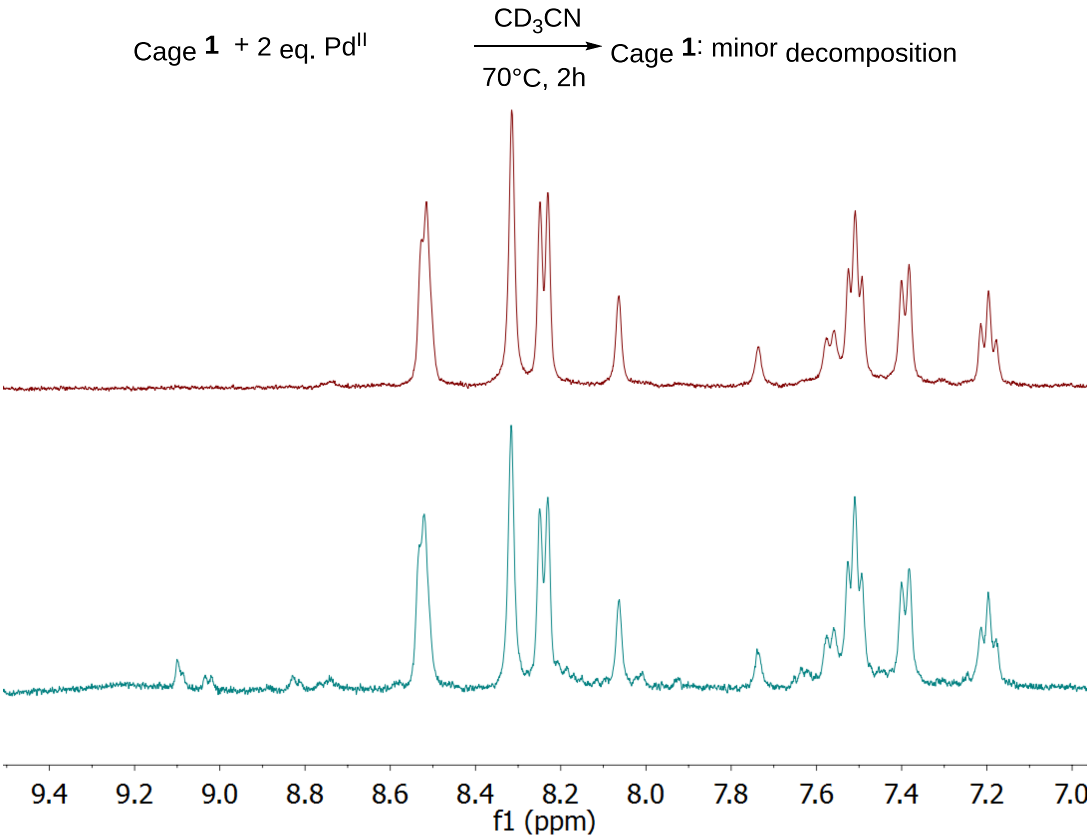

Figure S42. ^{1}H NMR spectrum of cage **1** in CD_3CN (top) and after the addition of 2 eq. of $[Pd(CH_3CN)_4](BF_4)_2$ and heating at 70 ℃ for 2 h to fully equilibrate the sample.

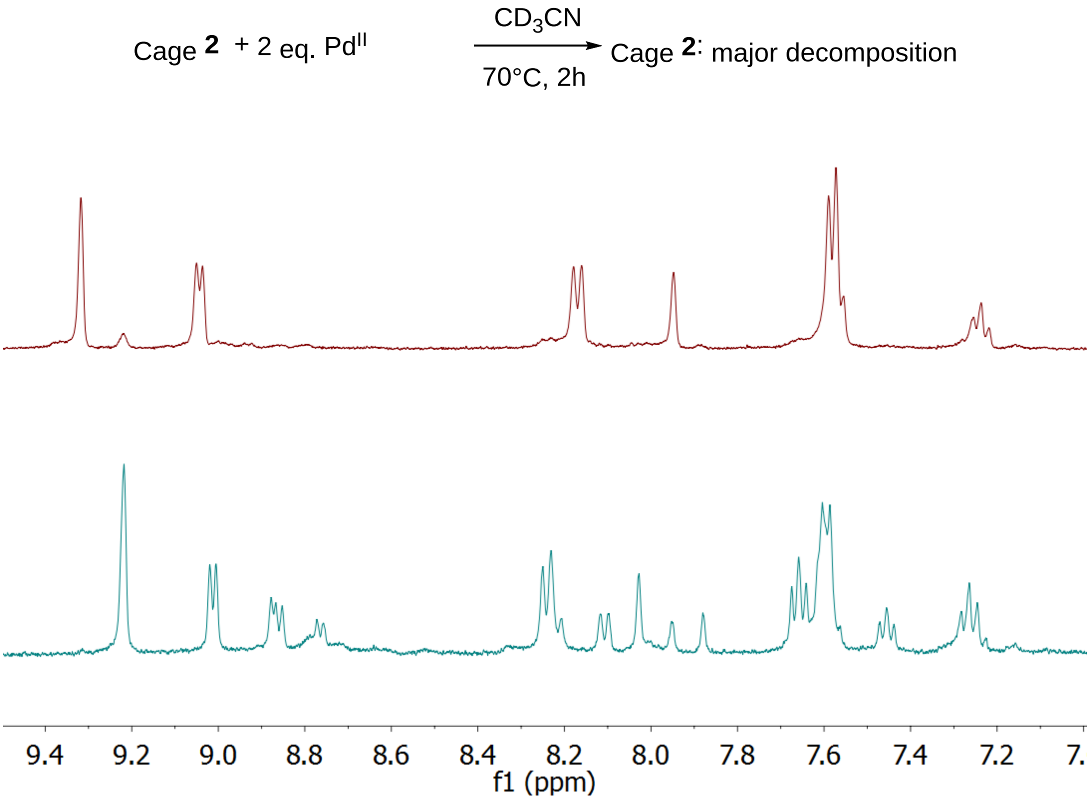

Figure S43. ^{1}H NMR spectrum of cage **2** in CD_3CN (top) and after the addition of 2 eq. of $[Pd(CH_3CN)_4](BF_4)_2$ and heating at 70 °C for 2 h to fully equilibrate the sample.

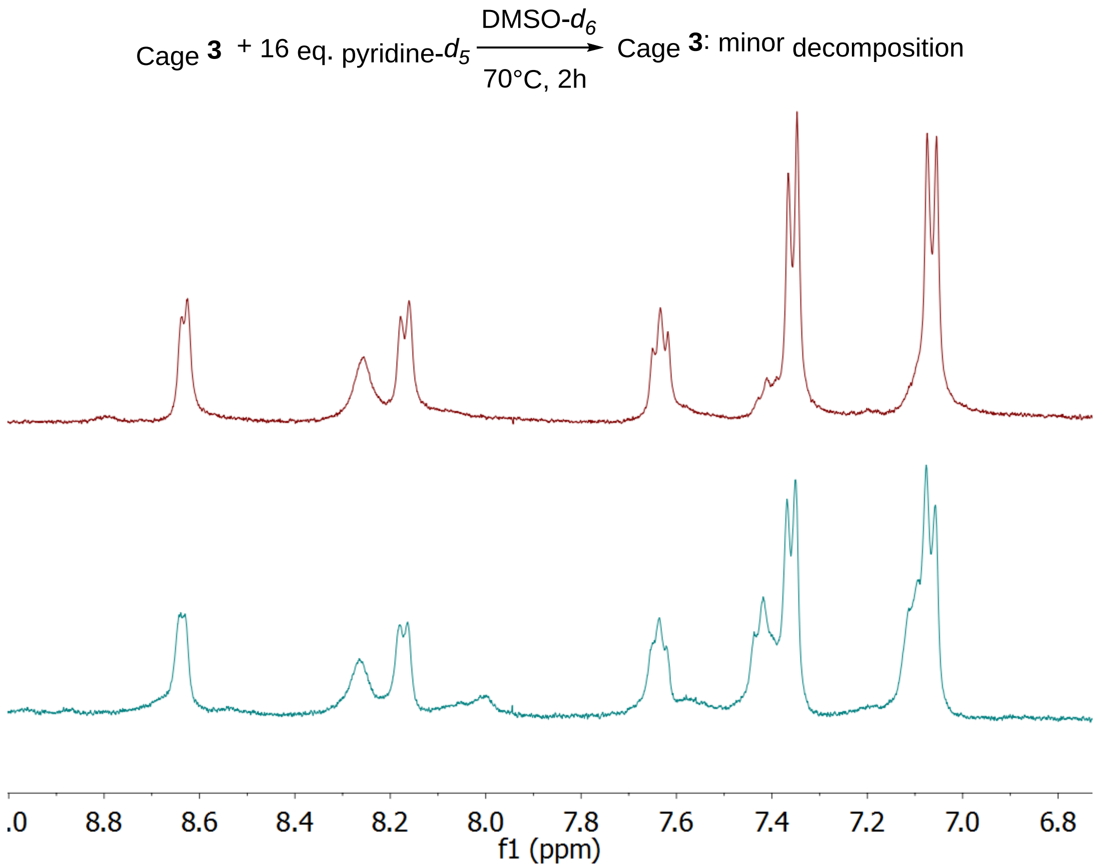

Figure S44. ^{1}H NMR spectrum of cage **3** in DMSO-d_6 (top) and after the addition of 16 eq. of pyridine-d_5 and heating at 70 °C for 2 h to fully equilibrate the sample.

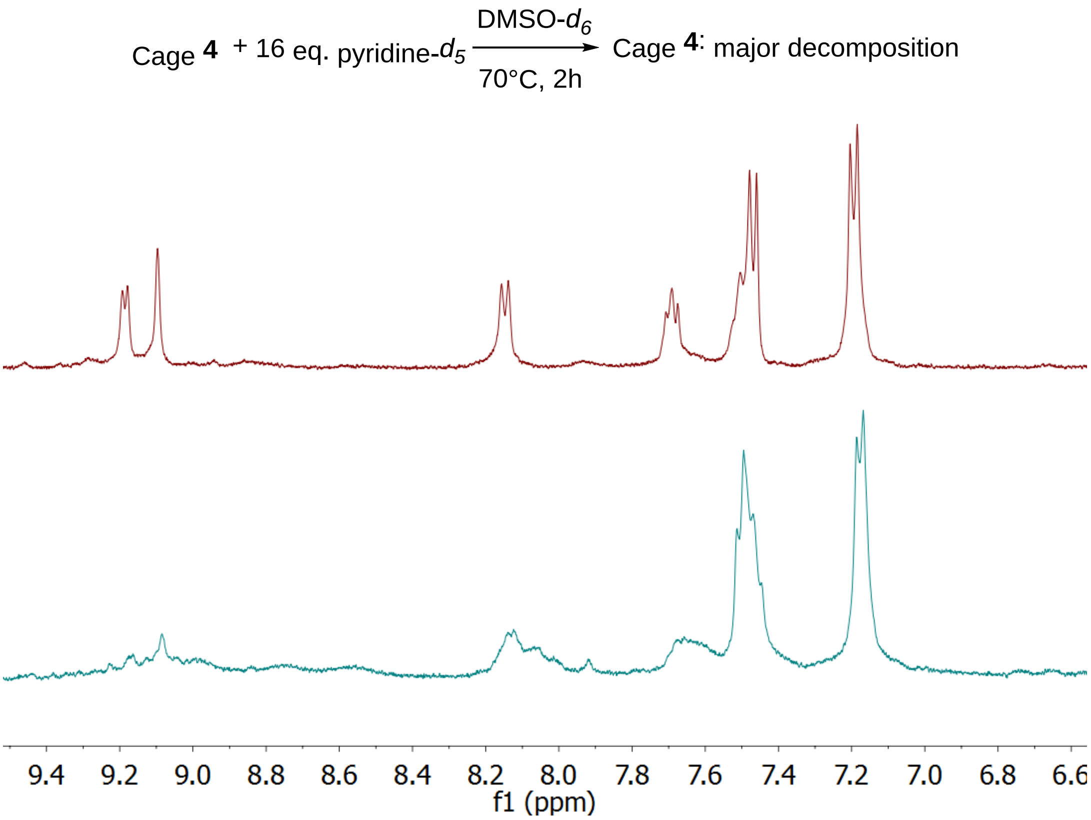

Figure S45. ^{1}H NMR spectrum of cage **4** in DMSO-d_6 (top) and after the addition of 16 eq. of pyridine-d_5 and heating at 70°C for 2 h to fully equilibrate the sample.

6. Single crystal X-ray analysis

Single crystals of sufficient quality for X-ray analysis were obtained by using slow diffusion with the following solvents:

L1 and **L2** DCM and diethyl ether

Cage **1** CH_3CN and diethyl ether

Cage **2** 20% CH_3CN in DMSO and dietheyl ether

Cage **3** 20% CH_3CN in DMSO and isopropylether

Cage **5** 20% CH_3CN in DMSO and isopropylether

Cage **6** 20% CH_3CN in DMSO and diethylether

Intensity data for all ligands and cages were collected on a Rigaku SuperNova dual system in combination with an Atlas CCD detector using Cu-$K_\alpha$radiation (λ = 1.54178 Å) at 140.0(2) K. The solutions were obtained by *SHELXT*[S3]; and the refinements were carried out by *SHELXL-2014*[S4] and OLEX2[S5] programs. The crystal structures were refined using full-matrix least-squares based on F^2 with anisotropically refined non-hydrogen atoms (except some disordered -nioxime fragments and solvent molecules which were refined in isotropic approximation). The anions in cages (all disordered and some ordered ones) were refined isotropically with U_{iso} and B-F and B...B distances fixed. Hydrogen atoms were placed in calculated positions by means of the "riding" model. Additional electron density found in the difference Fourier map of cage **1-3, 5**, **6** was treated by the SQUEEZE algorithm of *PLATON*[S6] and refined using ABIN instruction because of presence of a twinned component. Unfortunately, weak reflection ability and presence of a twinned component resulted in poor convergence factors for **2** and **5**. Nevertheless, the quality of the data is clearly sufficient to establish the connectivity of these structures. Intense disorder affected solvent molecules of **L1**, **L2**, **1, 3, 6** and several moieties of crystal structures **1, 2** and **5** tough restraints/constraints (involving SHELX commands: DFIX, SADI, SIMU, RIGU, EADP and ISOR) were used to handle it. Crystallographic data have been deposited with the CCDC no. 1511090–1511096. Copies of the data can be obtained free of charge on application to the CCDC, 12 Union Road, Cambridge, CB2 1EZ, U.K. (fax, (internet.) +44-1223-336033) or via https://summary.ccdc.cam.ac.uk/structure-summary-form.

Table S3. Crystallographic data for the metalloligands **L1** and **L2**.

Structure, CCDC no.	Ligand L1, 1511090	Ligand L2, 1511091
Empirical formula	$C_{56}H_{68}B_4Cl_8Fe_2N_{14}O_{12}$	$C_{43}H_{54}B_4Cl_6Fe_2N_{14}O_{12}$
Mol. weight / g mol^{-1}	1567.78	1326.64
Temperature / *K*	140.0(2)	140.0(2)
Wavelength / Å	1.54178	1.54178
Crystal system	Monoclinic	Triclinic
Space group	$P2_1/c$	$P\overline{1}$
a / Å	19.4453(5)	8.4174(8)
b / Å	15.7177(2)	19.6607(14)
c / Å	23.5873(7)	20.1792(15)
α / °	90	60.961(8)
β / °	109.174(3)	79.048(8)
γ / °	90	87.787(7)
Volume / Å^3	6809.2(3)	2860.9(5)
Z	4	2
Density / g cm^{-3}	1.529	1.540
Absorption coeff. / mm^{-1}	6.887	7.241
Crystal size / mm^3	0.74 x 0.11 x 0.10	0.31x 0.12x 0.09
Θ range / °	3.44 to 76.19	4.43 to 76.08
Reflections collected	50099	20081
Independent reflections	14020 [*R*(int) = 0.056]	11426 [*R*(int) = 0.066]
Observed reflections	10452	8126
Completeness	99.7 % (to Θ = 67.7°)	99.6 % (to Θ = 67.68°)
Absorption correction	Semi-empirical from equivalents	Semi-empirical from equivalents
Max. & min. transmission	0.62 and 0.16	0.75 and 0.48
Data / restraints / parameters	14020 / 6 / 858	11426 / 15 / 741
Goodness-of-fit on F^2	1.07	1.07
Final *R* indices [I > 2 s (I)]	*R*1 = 0.085, w*R*2 = 0.176	*R*1 = 0.081, w*R*2 = 0.175
R indices (all data)	*R*1 = 0.109, w*R*2 = 0.189	*R*1 = 0.110, w*R*2 = 0.194
Extinction coefficient	-	-
Larg. diff. peak/hole / eÅ^{-3}	2.12 and -2.65	1.12 and -0.93
Flack x (Parsons)	-	-

Table S4. Crystallographic data for the coordination cages **1-3**, **5**, **6**.

**Structure**	**1**	**2**	**3**	**5**	**6**
CCDC no.	**1511092**	**1511093**	**1511094**	**1511095**	**1511096**
Empirical formula	$C_{226}H_{267}B_{20}F_{16}Fe_8N_{65}O_{48}Pd_2$	$C_{160}H_{192}B_{20}F_{16}Fe_8N_{56}O_{48}Pd_2$	$C_{262}H_{304}B_{20}F_{16}Fe_8N_{64}O_{50}Pd_2S_{0.5}$	$C_{244}H_{264}B_{20}F_{16}Fe_8N_{56}O_{56}Pd_2$	$C_{264}H_{306}B_{20}F_{16}Fe_8N_{62}O_{56}Pd_2$
Mol. weight / g mol^{-1}	5841.83	4847.48	6345.5	6056.90	6423.49
Crystal system	Triclinic	Monoclinic	Triclinic	Triclinic	Tetragonal
Space group	$P\bar{1}$	$P2_1/c$	$P\bar{1}$	$P\bar{1}$	*P4/mnc*
a / Å	20.2193(12)	41.2884(9)	21.3764(8)	20.6996(19)	17.54710(8)
b / Å	20.6255(11)	14.0841(4)	21.4422(9)	21.9894(10)	17.54710(8)
c / Å	21.5175(12)	54.0058(15)	23.5362(10)	23.0916(12)	62.6742(5)
α / °	63.610(5)	90	105.440(4)	96.416(4)	90
β / °	76.958(5)	97.789(2)	91.132(3)	91.400(6)	90
γ / °	85.618(5)	90	90.267(3)	109.195(6)	90
Volume / Å^3	7828.1(8)	31115.2(14)	10396.1(7)	9843.2(12)	19297.4(2)
Z	1	4	1	1	2
Density / g cm^{-3}	1.239	1.068	1.014	1.022	1.105
Absorption coeff. / mm^{-1}	4.465	4.396	3.422	3.576	3.678
Crystal size / mm^3	0.37 x 0.13 x 0.07	0.53 x 0.07 x 0.05	0.45 x 0.22 x 0.19	0.36 x 0.29 x 0.18	0.18 x 0.14 x 0.09
Θ range / °	3.24 to 62.05	3.24 to 51.14	3.82 to 76.74	3.60 to 76.93	3.29 to 75.65
Reflections collected	46789	144401	78249	74423	140802
Independent reflections	23977 [R(int) = 0.051]	33180 [R(int) = 0.128]	41971 [R(int) = 0.054]	39518 [R(int) = 0.099]	10092 [R(int) = 0.033]
Observed reflections	17759	18348	28354	18393	9604
Completeness	97.2 % (to Θ = 65.0°)	98.7 % (to Θ = 51.14°)	99.8 % (to Θ = 67.5°)	99.8 % (to Θ = 67.5°)	99.8 % (to Θ = 67.67°)
Max. & min. transmission	0.74 and 0.29	0.75 and 0.48	0.20 and 0.04	0.64 and 0.39	0.78 and 0.66
Data / restraints / parameters	23977 / 89/ 1683	33180 / 647 / 2734	41971 / 119/ 1998	39511 / 71 / 1697	10092 / 53 / 495
GOF	1.00	0.99	1.02	1.07	1.04
Final R indices [I > 2 s (I)]	$R1$ = 0.096, w$R2$ = 0.206	$R1$ = 0.147, w$R2$ = 0.291	$R1$ = 0.098 w$R2$ = 0.209	$R1$ = 0.150, w$R2$ = 0.291	$R1$ = 0.065, w$R2$ = 0.165
R indices (all data)	$R1$ = 0.121, w$R2$ = 0.222	$R1$ = 0.213, w$R2$ = 0.332	$R1$ = 0.132, w$R2$ = 0.227	$R1$ = 0.229, w$R2$ = 0.336	$R1$ = 0.067, w$R2$ = 0.167
Larg. diff. peak/hole / eÅ^{-3}	2.12 and -2.65	3.38 and -1.93	1.11 and -2.07	2.28 and -3.57	1.40 and -2.21

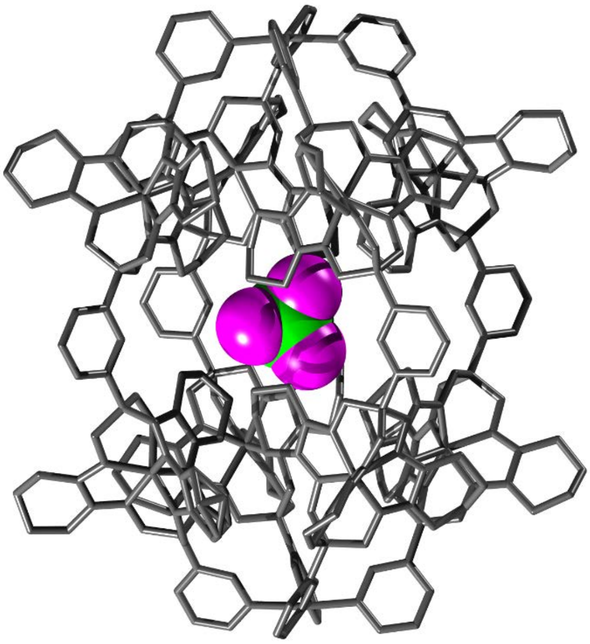

Figure S46. Molecular structures of cage **1** with space filling representation of the encapsulated BF_4^- anion. Hydrogen atoms are omitted for clarity. Green: B; pink: F; grey: all other atoms.

7. References

S1. J. M. Tour, A. M. Rawlett, M. Kozaki, Y. Yao, R. C. Jagessar, S. M. Dirk, D. W. Price, M. A. Reed, C.-W. Zhou, J. Chen, W. Wang and I. Campbell, *Chemistry – A European Journal*, 2001, **7**, 5118-5134.

S2. L. Patiny and A. Borel, *Journal of Chemical Information and Modeling*, 2013, **53**, 1223-1228.

S3. G. M. Sheldrick, *ActaCryst., Sect. A.* **2015**, *A71*, 3-8.

S4. G. M. Sheldrick, *ActaCryst., Sect. C.* **2015**, *C71*, 3-8.

S5. O. V. Dolomanov, L. J. Bourhis, R. J. Gildea, J. A. K. Howard, H. Puschmann, *J. Appl. Cryst.* **2009**, *42*, 339-341.

S6. A. L. Spek, *ActaCryst., Sect C.* **2015**, *C71*, 9-18.
